# Fingerprinting, structure, and genetic relationships among selected accessions of blue honeysuckle (*Lonicera caerulea* L.) from European collections

**DOI:** 10.1016/j.btre.2022.e00721

**Published:** 2022-03-23

**Authors:** Miłosz Smolik, Ireneusz Ochmian, Aleksandra Bobrowska-Chwat, Gerard Chwat, Liina Arus, Piotr Banaszczak, Jan Bocianowski, Paweł Milczarski, Krystyna Ostrowska

**Affiliations:** a Department of Plant Genetics, Breeding and Biotechnology, West Pomeranian University of Technology in Szczecin, Słowackiego 17, 71-434 Szczecin, Poland; b Department of Horticulture, West Pomeranian University of Technology in Szczecin, Słowackiego 17, 71-434 Szczecin, Poland; c Department of Plant Protection, West Pomeranian University of Technology in Szczecin, Słowackiego 17, 71-434 Szczecin, Poland; d Polli Horticultural Research Centre, Institute of Agricultural and Environmental Sciences, Estonian University of Life Sciences, Kreutzwaldi 1a, 51006 Tartu, Estonia; e Arboretum SGGW, Rogów Forestry Experimental Station, Warsaw University of Life Science – SGGW, Leśna 5b, 95-063 Rogów, Poland; f Department of Mathematical and Statistical Methods, Poznan University of Life Sciences, Wojska Polskiego 28, 60-637 Poznań, Poland

**Keywords:** *Lonicera*, R-ISSR, New molecular markers, STRUCTURE, MDS, PCoA

## Abstract

Due to its value and economic importance, the genome of *Lonicera caerulea* L. has been widely studied in various fields of science. In this study the genetic structure and relationships between 24 accessions of *L. caerulea* of different origins were assessed. A total of 692, 814, and 258 loci were amplified using 43 RAPD (random amplified polymorphic DNA), 40 ISSR (intersimple sequence repeat), and 20 R-ISSR (RAPD+ISSR) primers, respectively. Among the amplified loci, 66–78% were polymorphic and 12–20% were private. Selected R-ISSR sequences were detected in *Lonicera japonica* transcripts. Cluster and STRUCTURE analyses performed for each of the techniques revealed the existing differences and unknown similarities between the genotypes. The *r*-factor values calculated in the Mantel test indicated highly significant positive correlations between the Nei distance matrices, similar to the *F*_ST_ values (*F*_ST_RAPD_ = 0.223, *F*_ST_ISSR_ = 0.279, *F*_ST_R-ISSR_ = 0.363) determined in the analysis of molecular variance. It was found that 78%, 72%, and 64% of the genetic variations were related to the differences observed within the populations, which suggest that the variations are mainly reflected in the differences among the genotypes. The principal coordinate analysis showed greater differences between the mean distances of the *Lonicera* genotype pair and the actual distances of the same pairs on the Nei matrix compared to multidimensional scaling. These differences were 45%, 56%, and 42% higher for RAPD, ISSR and R-ISSR, respectively.

## Highlights

• Scarcely on December 13, 2018, *L. caerulea* fruits were included in the list of novel foods in EU.
• Hence, the growing interest in *L. caerulea* extends to its genome.
• R-ISSR explores other than the RAPD, ISSR, AFLP, and RFLP techniques part of genome and analyze other range of genetic variability.
• R-ISSR markers could be used for *Lonicera* core germplasm collection, development of SCARs, genetic map construction, barcoding, protection of variety rights, MAS, and genomic selection.

## 1 Introduction

The genus *Lonicera* L. belongs to the *Caprifoliaceae* Juss. Family. It includes about 180 species, of which many are used as ornamental, medicinal, and edible shrubs. The fruits produced by edible shrubs are known by different names, such as blue honeysuckle, haskap, haskap berry, honeyberries, or zimolost (in Russian). Among the species of *Lonicera*, blue honeysuckle (*Lonicera caerulea* L. sensu lato) is the most widespread with a circumboreal distribution, ranging from North America and mountains of South Europe through the Middle East and Northern Asia to Japan [1, 12, 23, 35, 46].

Farmers, breeders, and scientists have been showing an increased interest in investigating the biological diversity of various genotypes of blue honeysuckle. This is due to its many desired and unique traits, including tolerance to low temperatures (even below −40 °C), different sizes, shapes, colors, and weight of fruits, ease of processing, and above all, the high content of macro- and microelements, flavonoids, phenolic acids, ascorbic acid, anthocyanins, proanthocyanidins, and other active substances [57, [66], [67], [68]].

Blue honeysuckle fruit are used to treat fever, headache, urinary disorders, diabetes mellitus, and rheumatoid arthritis. Moreover, the chemical compounds found in these fruit aid in the reduction of blood pressure and preventing osteoporosis and anemia, besides their antifungal, anticancer, antiadherence, antiviral, and chemoprotective effects [2, 3]

The genotypes of blue honeysuckle have been included in *Isika* (Adans.) Rehder section and *Caeruleae* Rehder subsection [1, 12]. The number of *Lonicera* spp. included under the *Caerulea* subsection ranges from 1 to as many as 17 (19) [4, 5]. However, it is still unclear whether they should be considered as subspecies, varieties, forms, or as a separate group of *Lonicera* spp. [6], [7], [8], [9]. Most *Lonicera* spp. are diploids (2n = 2x = 18), but belong to the *Caeruleae* subsection and are predominated by tetraploids (2n = 4x = 36) [6, [9], [10], [11], 46]. Some exceptions have also been reported. Miyashita et al. [13] showed that Japanese populations of *L. caerulea* L. included diploids. The disagreement with regard to the taxonomy of the subsection *Caeruleae*, although valid, gives rise to derivative problems. Naugžemys et al. [9] stated that *Lonicera kamtschatica* can be regarded as a separate species, subspecies, or variety. This is quite similar to using different names for the same cultivar. For example, the Sinyaya Ptitsa cultivar is called Blue Bird, Zolushka as Cinderella, Tomichka as Blue Belle, and so on [14].

*Lonicera caerulea* L. was first introduced to Poland from Russian collections (*L. caerulea* ssp. *edulis* and *kamtschatica*) around 3 decades ago. Later, many valuable cultivars were bred in several centers in the country. Until 2020, the species *L. caerulea* L. was not mentioned in the Polish legislation's list of plant species whose cultivars are subject to registration and whose seed material can be produced, assessed, and controlled. As a result, the genotypes (cultivars) of blue honeysuckle were not included in the National Register (KR) and were not officially controlled and protected. Thanks to recent taxonomic rearrangements, blue honeysuckle has been recognized as a “traditionally grown” species in Poland, according to Art. 6 of the Seed Act (Journal of Laws from 2021, item 129) and its cultivars were legally registered. To date, there are three known cultivars, namely Aurora, Honey Bee, and Siniy Utes, of which Siniy Utes has exclusive cultivar rights. Two other cultivars (Nowa Kamczacka and Lawina) have also been submitted for rights.

The growing interest in *L. caerulea* L. extends to its genome. Molecular techniques, including those based on arbitrarily amplified markers, have provided strategies for developing marker systems that can detect DNA variations and hence can be useful for phylogenetic studies, barcoding, origin verification, protection of cultivar rights, and in MAS [15]. Most dominant markers are generated at random across the genome. For *L. caerulea*, RFLP (restriction fragment length polymorphism) [16, 17], RAPD (random amplified polymorphic DNA) [18, 19], ISSR (intersimple sequence repeat) [20, 21], and AFLP (amplified fragment length polymorphism) [22] have been identified. In addition, ITS (Internal Transcribed Spacer) polymorphism [17] and chloroplast DNA noncoding regions were described for this species [22]. The advantages and limitations of RAPD, ISSR, and AFLP have already been explained by several authors, including Williams et al. [24], Zietkiewicz et al. [25], and Vos et al. [26]. One of the main limitations of dominant markers is their inability to distinguish dominant homozygotes from heterozygotes. Simmonsa et al. [27] and Poczai et al. [15], scrupulously pointing out the disadvantages of arbitrarily amplified DNA (ADD) markers, emphasized the possibility of their application in preliminary studies on the genomes of little-known plants, such as *L. caerulea.* On December 13, 2018, *L. caerulea* fruits were included in the list of novel foods (2015/2283 EU), which allowed the legal marketing of blue honeysuckle berries in the European Union (EU).

Given the scarcity of biological information, the use of techniques that do not require sequence knowledge appears justified. According to Poczai et al. [15], understanding the limitations of dominant markers may enable their appropriate use and a shift toward more restricted ones, such as from ISSR (dominant) to SSR (co-dominant) markers [28] or gene-targeted (GTMs) and functional markers (FMs) [29].

The primary goal of both genetic and breeding studies is to determine the genetic structure of a population. The *F*-statistics (analysis of molecular variance, AMOVA) used for this purpose require *a priori* knowledge of the structure of the studied population. Clustering analyses can be used to understand the population structure [30]. According to Culley et al. [31], AMOVA was not originally developed for the analysis of purely dominant data, and the data must be presently treated as molecular haplotypes. Starting with an analysis of differences in the genetic variant distribution among populations, Pritchard et al. [32] implemented a Bayesian iterative algorithm in STRUCTURE, placing the samples into groups, the members of which had similar patterns of variations. Moreover, STRUCTURE version 2.2 and above include a model dealing with the genotypic ambiguity associated with dominant markers, such as with Ploidy>2, which is highly valuable in research on tetraploids.

The aim of the present study was to determine the scope of genotypic variability, mutual genetic relations, population structure between and within selected accessions from Polish, Estonian, Russian, and Ukrainian collections using RAPD, ISSR, and R-ISSR (RAPD+ISSR) techniques. This study is the first to use R-ISSR for the *Lonicera* genome. We have demonstrated the usefulness of this technique for genetic profiling, identification of cultivar (molecular) markers, and construction of genetic maps.

## 2 Materials and methods

Plant research material of *Lonicera* sp. were obtained as cultivars and clones from different European collections. His-donors and information about origin has been presented in Table 1. Genotypes of L7661, L7662, L7987, DLN, SIN, ATU, CZA, WOJ, ZIE and JOL have been used earlier in preliminary work to describe their relationships using ISSR [20].

**Table 1:** Characteristic of plant material of *Lonicera caerulea* L.

Genotypes and abbreviations^1^		Donor	(O)rigin/(B)reeder
L. *caerulea* L. 7661	L7661	Arboretum in Rogów, Forest Experimental Station, Warsaw University of Life Sciences Warsaw, Poland A-FES WULS	The Conservatory and Botanical Garden of the City of Geneva, Switzerland (O)
L. *caerulea* var. *edulis* Turcz. ex Herder 7662	L7662		Jakutsk Hortus Botanicus, Russia, Jakutsk (O)
L. *caerulea* L. 7987	L7987		The Conservatory and Botanical Garden of the City of Geneva (O)
Brązowa	BRA	‘Jagódka' Zygmunt Landowski Lębork, Poland ZL	‘Jagódka' Zygmunt Landowski Lębork, Poland (B)
Czarna	CZA		
Zielona	ZIE		
Wojtek	WOJ		Zofia and Hieronim Łukaszewscy Osielsko, Poland (B)
Atut	ATU	Zofia and Hieronim Łukaszewscy Osielsko, Poland ZHŁ	Zofia and Hieronim Łukaszewscy Osielsko, Poland (B)
Duet	DUE		
Jolanta	JOL		
Clone 22	C22		
Clone 38	C38		
Clone 44	C44		
Clone 46	C46		
Mińsk	MIN		The plant brought by Ms Zofia Łukaszewska from the vicinity of Minsk, Belarus (O)
Dlinnopłodna	DLN	Ukrainian National Forestry University, Lviv, Ukraine UNFU	Russia (O)
Sineglazka	SIN		
Wołoszebnica/Volshebnica^2^	WOL		
Goluboye Vereteno	GOL	Estonian University of Life Sciences, Polli Horticultural Research Center, Tartu, Estonia EULS	Bakcharskii Agricultural Station of the M.A. Lisavenko. Siberian Horticultural Research Institute Tomsk, Russia (B)
Herdi	HER		unknown
Iskra	ISK		unknown
Roksana	ROK		Bakcharskii Agricultural Station of the M.A. Lisavenko. Siberian Horticultural Research Institute Tomsk, Russia (B)
Sinyaya Ptitsa/Blue Bird^2^	SPT		
Zolushka/Cinderella^2^	ZOL		

1 abbreviations of the names of genotypes presented in the experiment.

2 replaced names used in the scientific literature.

**DNA preparation.** Genomic DNA was prepared from leaves collected in early spring from bushes of the above-mentioned collections. The DNA preparation was carried out with a DNeasy Plant Mini Kit (Qiagen). DNA concentration and purity was determined spectrophotometrically (Epoch–BioTek, USA). Amplifications were conducted in a Mastercycler 5333 thermal cycler (Eppendorf, Germany).

**RAPD.** RAPD amplifications were performed with the use of pre-selected 50 decamer primers (University of British Columbia, Canada). Amplifications were carried out according to the protocol presented by Williams et al. [24].

**ISSR.** A fifty pre-selected microsatellite ISSR primers, differing in repeated sequence motifs and quality of nucleotides both at the 3′- and 5′-end were used for amplification. They were designed in the University of British Columbia (Canada), set#9, and synthesized in Sigma-Aldrich (Poland). Primer marked with an asterisk are blackened from various publications. Their sequences are listed under Table S1. Amplifications were carried out according to the protocol presented by Ziętkiewicz et al. [25].

**R-ISSR.** The genetic profiles were obtained as a result of using a combination of RAPD and ISSR primers for amplification. They were selected in preliminary analyses according to the methodology presented by [33, 34], and [36, 37].

**Electrophoresis.** RAPD, ISSR and R-ISSR amplification products were mixed with 6 × Orange Loading Dye Solution and analyzed with electrophoresis (2% agarose gel – Basica LE GQT, Prona, Spain) in 1 × TBE buffer in SubCell GT cells (BioRad) at 100 V for 2 h. Separations were made with 100 bp GeneRuler Plus DNA Ladder (Thermo Scientific). Gels were stained with ethidium bromide, visualized (G:Box/GeneSnap - Syngene, USA) and scored for band presence (1) or absence (0) using Diversity One 1.3 (Pharmacia Biotech, USA). Number and character of amplified loci was determined with respect to each examined *Lonicera* sp. genotype.

**PCR product extraction** from agarose gel was made for selected amplicons with a QIAquick Gel Extraction Kit (Qiagen, Hilden, Germany) according to the manufacturer's protocol.

**TOPO TA Cloning** extracted products were cloned into pCR2.1 vectors with a TOPO TA cloning® kit (Invitrogen), and then transformed into competent *E. coli* TOP10 cells according to the manufacturer's protocol. Selection of transformants was made on the basis of colony color assessment (white-blue). DNA plasmids were isolated from 2 ml of liquid LB medium with a Plasmid Midi kit (A&A Biotechnology, Poland). Insert sizes were assessed after 5-minute digestion (*Eco*RI – fast digest) (Thermo Scientific) and separation in 1% agarose gel.

**Sequencing and sequence analysis** was conducted with a Beckman Coulter DTCS kit and a CEQ 8000 machine – capillary sequencer according to the manufacturer's manual. Data was analyzed with BioEdit application http://www.mbio.ncsu.edu/BioEdit/bioedit.html). The analysis of selected sequences, including in particular R-ISSR, was performed to answer two questions whether: (i) the products amplified as R-ISSR, are heteroamplicons, (ii) there are exist in official databases similar annotations. There were compared using the Basic Local Alignment Search Tool (BLAST) of the NCBI and Chinese National GeneBank official databases (CNGB).

**Data analysis.** The DNA fingerprints were used to assess the genetic variability in the tested *Lonicera* genotypes. For each genotype, the presence (1) or absence (0) of amplicons was scored. The genetic distance matrix, which included the 24 accessions of *Lonicera* sp*.*, was calculated using Nei and Li's [52] coefficient, while the genetic relationships between the genotypes were determined by generating dendrograms using the neighbor-joining method. The strength of the internal branches was tested by bootstrap analysis using 2000 pseudoreplications. All analyses were carried out in TREECON 1.3 [53]. The genetic similarity matrices for RAPD, ISSR, and R-ISSR were generated using the PhylTool software [54] using Nei's and Jaccard's coefficients, respectively. The matrices were compared by calculating the product moment correlation (*r*_AB.C_) (Pearson) and Mantel (Daniel) test statistics (*Z*) of significance using the *XL*STAT software [55].

**Structure analysis**. The population structure and admixed individuals were analyzed and individuals were properly assigned to *Lonicera* populations using the Bayesian clustering software STRUCTURE v.2.3.4. [32]. This software was installed as a package with graphical front end and as a package without front end for LINUX on High-Performance Computing (HPC) cluster in University Information Technology Center (UITC) at the West Pomeranian University of Technology in Szczecin (WPUT, Poland). All simulations presented here were conducted using HPC. It consists of four computing nodes (server), each of which is equipped with 12 processor cores (2 × 6) with Hyper-Threading support and has 128 GB RAM. Red Hat Enterprise Linux Server v. 7.5 was used as the operating system. The most likely number of populations (*K*) was estimated under the admixture model, based on correlated allele frequencies, and due to the presence of dominant data, by setting RECESSIVEALLELES = 1. The number of presumed populations (*K*) was fixed from 1 to 10. Twenty independent runs were performed for each *K* with, for example, 1000,000 number of Markov Chain Monte Carlo (MCMC) iterations and a burn-in period of, for example, 100,000 lengths. Δ*K* method in Structure Harvester and Evanno correction were applied for determining the number of *K* [56, 58]. The STRUCTURE permutation results were calculated using CLUMPP [59], and the bar charts of the structure of the tested *Lonicera* accessions were plotted using Distruct [60]. Additional population substructure within clusters was tested as described by [48], and analyses were repeated for each inferred population cluster separately.

**AMOVA, MDS, and PCoA.** The hierarchical AMOVA was carried out using Arlequin 3.5.2.2. [61]. [31] reported that this method considers dominant data as haplotypes and divides the total variance into covariance components associated with the differences among individuals within populations, differences among individuals in different populations within groups, and differences among groups. Significance levels for variance component estimates were determined using the approach of 10,100 permutations. Graphs were generated using R [62] based on the output data of Arlequin 3.5.2.2. Starting with Nei's distance matrixes for RAPD, ISSR, and R-ISSR the following packages were used: SMACOF [63] to perform multidimensional scaling (MDS), stress, and bootstrap; APE [64] to perform principal coordinate analysis (PCoA) and stress; and MultBiplotR [65] to perform bootstrap for PCoA. In addition, after standardizing the Nei distance matrices, the differences between the mean distances of the *Lonicera* genotype pair in the MDS and PCoA plots and the actual distance of the same pair on the Nei matrices were calculated. Considering the relationship between MDS and PCoA, these differences were included in the charts.

## 3 Results

The examined accessions of *Lonicera* spp. exhibited considerable genetic variability (Table 1). Each of the genotypes was assigned a unique RAPD, ISSR, or R-ISSR fingerprint. The genotypes obtained from the A-FES WULS collection were genetically distinguished, and the differences in the gene pool that are characteristic of accessions or clones were determined based on their origin.

**Fig. 1:**
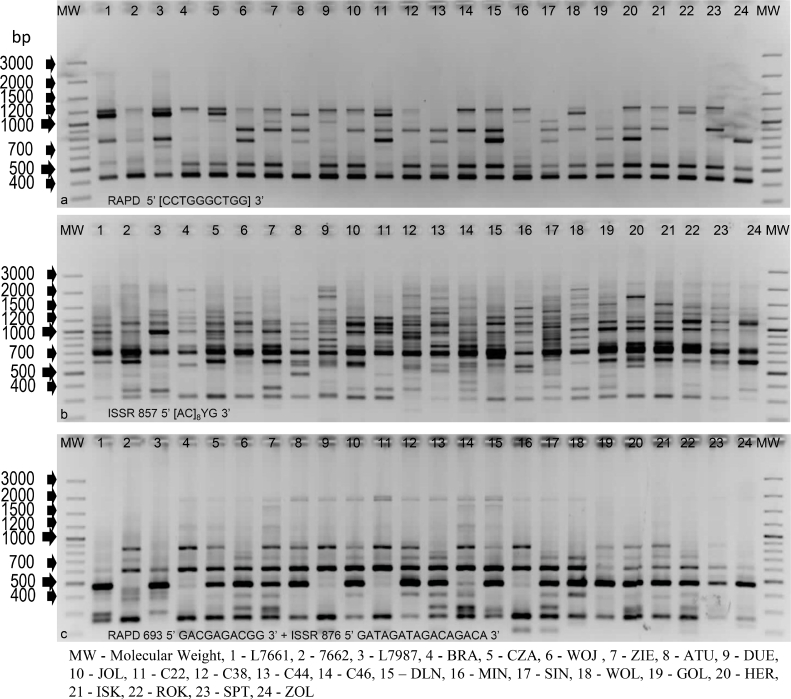
Electroforetic patterns of selected RAPD, ISSR and R-ISSR fingerprints for twenty four investigated *Lonicera caerulea* L. genotypes.

**RAPD.** Clear genetic profiles were obtained in amplifications with 43 out of 100 applied RAPD primers (Table 2, Fig. 1a). A total of 692 loci (6971 amplicons) were amplified for the 24 examined honeysuckle accessions. Among the amplified loci, 463 (67%) were polymorphic, 137 (20%) were private, and 92 (13%) were monomorphic (Table 2). The highest number of loci (34) was amplified with primer 24, and the lowest number (3) with primer 231. Each RAPD primer amplified on average 16 loci, which included 11 polymorphic, 2 monomorphic, and 3 private loci. The most number of polymorphic loci (25) were amplified with primer 24, and the least number (1 and 2) with primers 82 and 277, respectively. The most number of private loci (9) were amplified with primers 24 and 292, and the least number (1) with primers 60, 82, 85, 228, 234, 276, 285, 287, and 322. The results of the RAPD analysis are presented in detail in Table S1-2.

**Table 2:** Characteristics of loci amplified using RAPD, ISSR and R-ISSR methods.

	Primers number	Number of loci amplified	Total of amplicons generated	Loci		
				Monomorphic	Polymorphic	Private
RAPD	43	692	6971	92 (13%)	463 (67%)	137 (20%)
ISSR	41	814	8475	81 (10%)	639 (78%)	94 (12%)
R-ISSR	20	258	3190	44 (17%)	169 (66%)	45 (17%)

**ISSR.** Forty-one ISSR primers were used for obtaining fingerprints (Table S1) for *Lonicera* accessions (Table 2, Fig. 1b), which resulted in clear genetic profiles. A total of 814 ISSR loci (8475 amplicons) were amplified. Among them, 639 (78%) were polymorphic, 81 (10%) were monomorphic, and 94 (12%) were private loci (Table 2). Each ISSR primer amplified on average 20 loci, which included 16 polymorphic, 2 monomorphic, and 2 private. The most number of polymorphic products (36, 35, and 34) were observed in the genetic profiles generated using primers pr7, 808*, and pr8, respectively, and the least number (9) with primer 861. The most number of private loci (6 and 5) were amplified with primers 808* and pr7 and primers 854* and pr12, respectively.

**Fig. 2:**
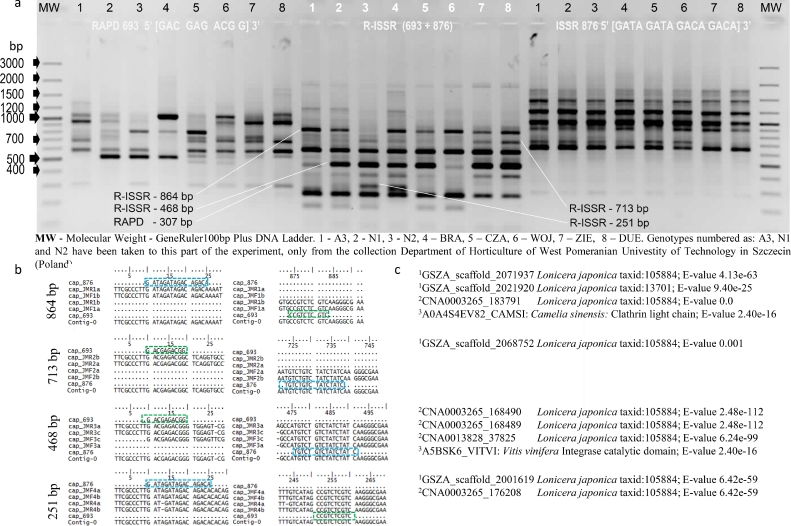
PCR products selected for sequencing. The primer sequences are marked in blue. China National GeneBank (CNGB): ^1^The 1000 Plants Project (oneKP), ^2^Medicinal Plants DataBase (MPDB); ^3^UniProt.

**R-ISSR.** The genetic variability of *Lonicera* accessions was determined by performing cyclically repeated polymerase chain reactions (PCRs) with 20 combinations of RAPD and ISSR primers selected in preliminary analyses (Table 2, Fig. 1c). The fingerprint for R-ISSR (i.e. after using the RAPD and ISSR primers) was found in one amplification, as shown in Fig. 2a. Shorter amplicons seen in the lower part of the electrophoregram resulted from the “shortening” of inter-microsatellite sequences by decamers (Fig. 2a). A total of 258 such generated R-ISSR loci were amplified (3190 amplicons), of which 170 (66%) were polymorphic, 44 (17%) were monomorphic, and 44 (17%) were private (Table 2). Each primer amplified on average 13 R-ISSR loci, which included 9 polymorphic, 2 monomorphic, and 2 private loci. The most number of polymorphic PCR products (12 for each) were found in the genetic profiles resulting from reactions with the primer combinations 615+811, 646+835, 693+835, and 818+876, and the least number of products (2) with primer combination 81+835. The most number (8) of private R-ISSR loci were amplified with primer combinations 615+811 and 211+876. Private loci were not amplified using six primer pairs. The results of the R-ISSR analysis are presented in detail in Table S1. Amplicons with lengths of 713 and 251 bp that migrated in agarose similarly to the respective ISSR and RAPD products in the R-ISSR profile were heteroamplicons (Fig. 2a). The heteroamplicon nature of two additional R-ISSR products (864 and 468 bp) was also confirmed by sequence analysis (Fig. 2b). For the sequences of four R-ISSRs, perfect matches were found in the databases oneKP, MPDB (China National GeneBank, CNGB), and UniProt (Fig. 2b and c).

**Fig. 3:**
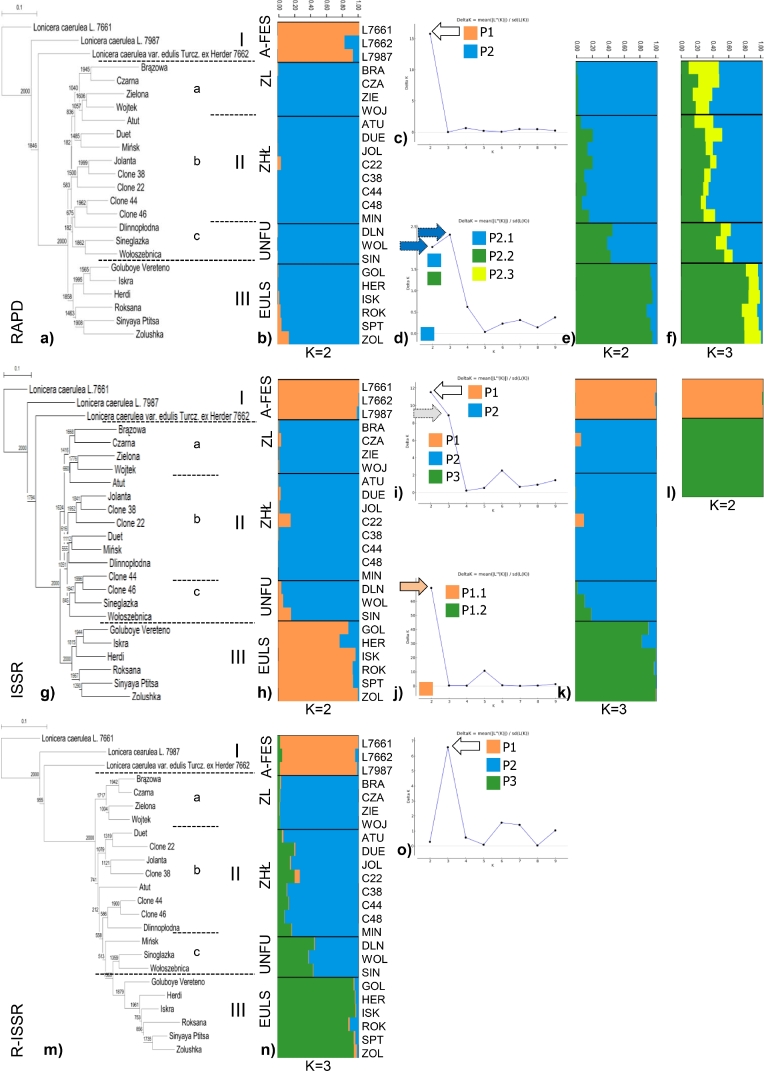
Results of the analysis performed using STRUCTURE. Each genotype is represented by a single vertical line assigned into different *K* colored segments with lengths proportional to each of the *K* inferred clusters. The composition of the P groups was presented in the text.

**GENETIC SIMILARITY ANALYSIS**. Nei's similarity coefficients for the examined genotypes were presented in the form of RAPD, ISSR, and R-ISSR matrices. The values of the coefficients ranged from 0.88 (ISK and GOL) to 0.38 (WOL and L7661) for RAPD, from 0.83 (GOL and ISK) to 0.34 (L7661 and L7662) for ISSR, and from 0.93 (C44 and C46) to 0.41 (L7662 and L7987) for R-ISSR. The information on Nei's and Jaccard's similarity coefficients for the examined accessions is provided in detail in Table S3. The topology of RAPD, ISSR, or R-ISSR dendrograms generally showed a similar grouping of genotypes depending on their origin/donors (Fig. 3a, g, and m). Species obtained from the Arboretum (A-FES WULS) and EULS collections exhibited different genotype grouping (groups I and III) compared to other accessions (Fig. 3a, g, and m). Same admixtures were especially observed for genotypes from ZL, ZHŁ, and UNFU collections (group II, a–c). Mantel test demonstrated highly significant positive correlations (*r*) for Nei's similarity matrices: *r*_RAPD-ISSR_ = 0.924 (*P* < 0.0001), *r*_ISSR-R-ISSR_ = 0.938 (*P* < 0.0001), and *r*_RAPD-R-ISSR_ = 0.929 (*P* < 0.0001). In addition, a highly significant correlation was observed between the matrices of RAPD, ISSR, and R-ISSR (*r*_RAPD ISSR R-ISSR_ = 0.411, *P* < 0.0001).

## BAYESIAN MODEL-BASED CLUSTERING FOR BINARY DATA SETS

**RAPD.** The Bayesian algorithm used in STRUCTURE revealed that two populations contained the studied genotypes of *Lonicera* sp. (Fig. 3b). For *K* = 2, we found the highest peak and Δ*K* value (Fig. 3c). Three genotypes from the A-FES WULS collection (12%) were included in the P1 population, while the remaining 21 (88%) from the ZL, ZHŁ, UNFU, and EULS collections were included in P2 (Fig. 3b). Within P2, 2–3 discrete populations (P2.1, P2.2 and P2.3) were found to exist (Fig. 3d). Analysis of the bar chart indicated the differences between ZL, ZHL, UNFU (P2.1), and EULS (P2.2) for *K* = 2 (Fig. 3e) and between UNFU, ZL+ZHL, and EULS for *K* = 3 (Fig. 3f). The UNFU genotypes were characterized by mixed ancestry P2.1–P2.3 (DLN 49+12+39%, WOL 43+13+44%, and SIN 53+11+36%). The existence of discrete populations was confirmed in additional simulations, with the use of 20 and 10 iterations, respectively, with a burn-in period of 50,000 lengths and 100,000 numbers of MCMC iterations (Fig. S1). At 20 iterations, the differences between Δ*K* = 2 and Δ*K* = 3 were small, which suggested the division into three populations (Fig. S1), while at 10 iterations Δ*K* = 3 (Fig. S1). Analysis of bar charts for other Δ*K* values indicated a visible separation between the genotypes from the UNFU and ZL+ZHL populations as well as between the ZL and ZHL populations (Fig. S1).

**ISSR.** Clustering using STRUCTURE grouped *Lonicera* genotypes in two populations, P1 and P2 (Fig. 3h). The highest peak and Δ*K* value were found for *K* = 2 (Fig. 3i). Nine genotypes (38%) from the A-FEE WULS (3) and EULS (6) collections were classified as P1, while the remaining 15 (62%) (ZL+ZHL+UNFU) were classified as P2 (Fig. 3h). A slight difference was found in the Δ*K* values for *K* = 2 and *K* = 3 (Fig. 3i), which led to the need for additional simulations. The existence of two discrete populations, P1.1 (AFES WULS) and P1.2 (EULS), was observed in P1 (Fig. 3j and l). Additional simulations confirmed the presence of discrete populations, as for RAPD markers (Fig. S1). Analysis of bar charts for *K* = 3 revealed a clear separation between the genotypes from the ZL+ZHL+UNFU and EULS populations, and with division into *K* = 6, also between the genotypes from UNFU, ZL, and ZHL (Fig. S1).

**R-ISSR.** After simulation with STRUCTURE using Evanno's correction, adopting *a priori* division into K from 1 to 10, the most probable population size (*K* = 3) was estimated based on the highest peak, for the value of Δ*K* (Fig. 3o). Twenty-one genotypes (88%) were found to belong to any one of the populations P1–P3 (Fig. 3n), while the remaining 3 (12%) seemed to have mixed ancestry. The genotypes from the A-FEE WULS collection belonged to P1. The P2 population included 15 genotypes from the Polish breeding (ZL, ZHŁ) and UNFU collections. The P3 population consisted of cultivars from the EULS collection (Table 3). Mixed ancestry (P2P3) was observed in the case of cultivars DLN (53+47%), WOL (60+40%), and SIN (55+45%). The results obtained with additional simulations from 20 to 10 iterations, with a burn-in period of 50,000 lengths and 100,000 numbers of MCMC replications after burn-in, respectively, are shown in Fig. S1. In both Bayesian simulations, the algorithm located the studied genotypes in three populations. Within P2 population, the subpopulations were clearly visible (Fig. S1).

**Table 3:** Summary analysis of molecular variance (AMOVA) for comparison among and within populations for three types of dominant markers.

Markers	Source of variation	Among populations	Within populations	Total
	d.f.	2	21	23
RAPD	Sum of squares	374.275	1385.767	1760.042
	Variance components	19.004	65.989	84.992
	Percentage of variation	22	78	
	F_ST_ = 0.22359, P (rand≥value) 0.00001			
ISSR	Sum of squares	657.575	1985.633	2643.208
	Variance components	36.743	94.554	131.297
	Percentage of variation	28	72	
	F_ST_ = 0.27984,P (rand≥value) 0.00001			
R-ISSR	Sum of squares	203.500	459.833	663.333
	Variance components	12.526	21.897	34.423
	Percentage of variation	36	64	
	F_ST_ = 0.36389, P (rand≥value) 0.00001			

Probability of obtaining a larger component estimate; number of permutations is equal to 10,100.

**Fig. 4:**
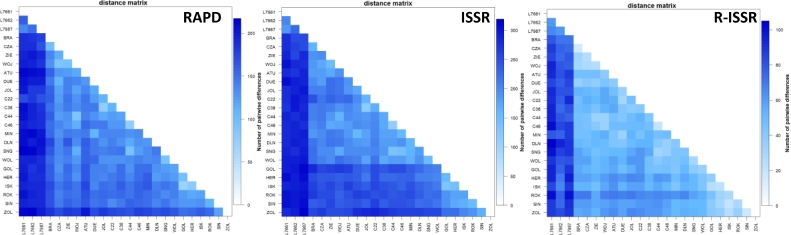
The number of molecular differences between all *Lonicera caerulea* L. genotypes studied.

**Fig. 5:**
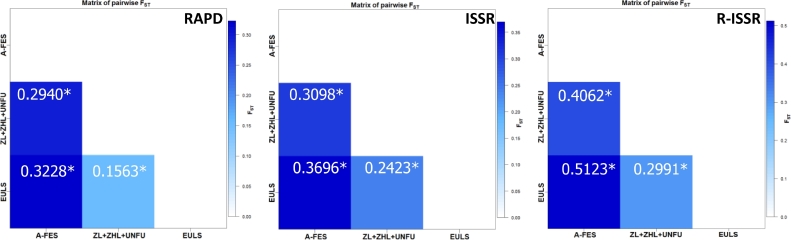
Matrix of pairwise F_ST_ values for population comparisons according to results of the STRUCTURE permutations. Intensity of blue color squares corresponds to the F values shown in the scale bar.

**Fig. 6:**
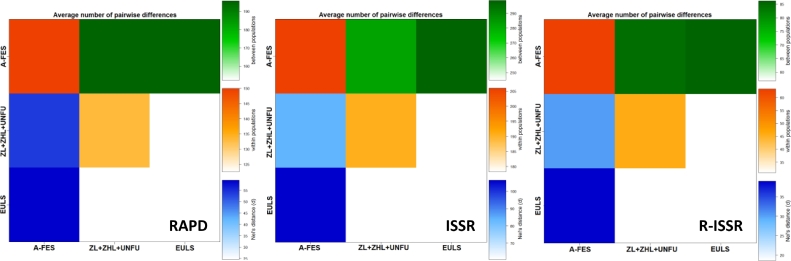
Matrix of average number of paired differences (π) between the *Lonicera* populations investigated. Orange on diagonal: within populations; green above diagonal: between pairs of populations presented between the groups, and blue below diagonal and Nei distance between populations.

**AMOVA.** This analysis was performed to evaluate the results of the STRUCTURE analysis (Table 3). It was observed that both intra- and interpopulation variability were significant (*P* < 0.001) for all datasets (Table 3). The variability among the populations ranged from 22% (for RAPD markers) to 36% (for R-ISSR markers), while variability within populations ranged from 64% (R-ISSR) to 78% (RAPD), which indicated that significant genetic variations existed within the populations. The results of the analysis of the sum of squares among (SSAP) and within (SSWP) the populations using three types of dominant markers are presented in detail in Table 3. The results of the investigation of genetic relationships between the studied *Lonicera* genotypes using RAPD, ISSR, and R-ISSR markers are shown in Table S2. The molecular differences between the studied *Lonicera* genotypes are graphically presented in Fig. 4. The pairwise F_ST_ values between populations (A-FES, ZL+ZHL+UNFU, EULS) were significant and ranged between 0.16 and 0.32 for RAPD, 0.24 and 0.37 for ISSR, and 0.30 and 0.51 for R-ISSR (Fig. 5). High pairwise differences were found between the *Lonicera* populations indicating their genetic diversity, and each of these populations showed significant within-population heterogeneity (Fig. 6).

**Fig. 7:**
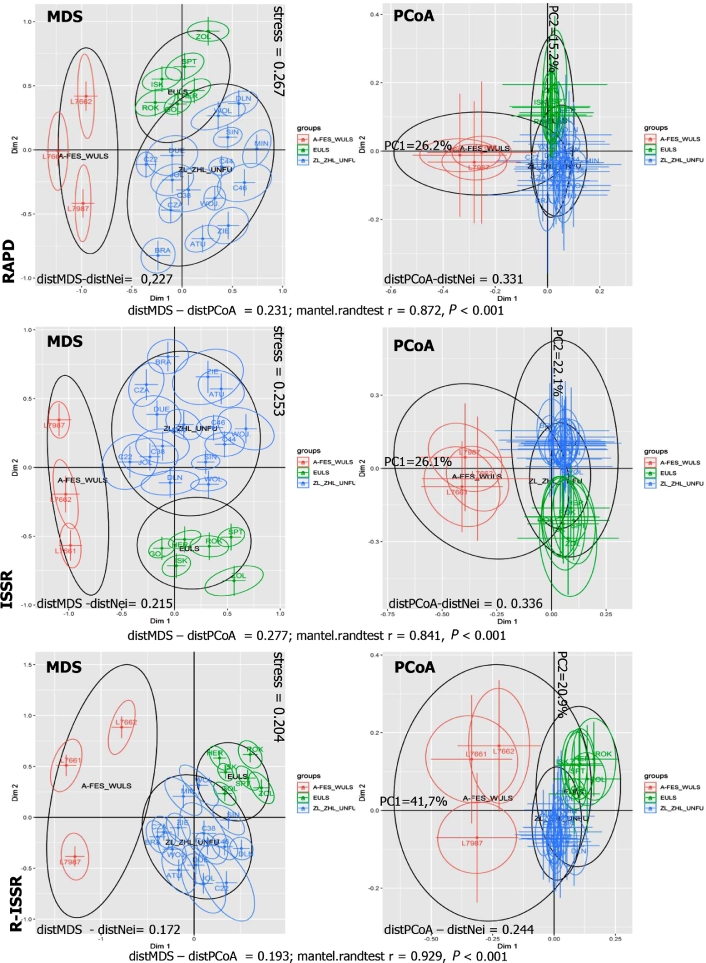
A two-dimensional plots of MDSs and PCoAs peformed for RAPD, ISSR and R-ISSR datasets using Nei's distance matrix. Upper right corners of MDSs plots stress values have been presented. The first and second principal coordinates account has been presented above main axex on PCoAs plots.Error bars correspond to the square root of the stress statistics. The bootstrap shows the sensitivity of the spatial configuration of the genotypes to the missing random loci in the dataset (500 alternative coordinates of the spatial configuration). The ellipses shown around the genotype (point) indicate the covariance of 500 coordinates, based on the assumption that the resulting alternative coordinates of the point configurations follow a 2D normal distribution. We expect that 60.65% of these coordinates fall within the ellipses. The co-variances of the alternative coordinates belonging to the groups designated by STRUCTURE are represented by black ellipses. This area contains 95% of the 500 bootstrap pseudo-replications for the designated groups.

**MDS and PCoA.** The results of these analyses are presented as two-dimensional (2D) plots in Fig. 7. They confirmed the division of the studied genotypes into three populations, in accordance with the dendrograms as well as the STRUCTURE bar charts (Fig. 3). For RAPD markers, the 2D PCoA plot showed that the first two principal components explained 26.2% and 15.2% of the total variation, respectively (Fig. 7). Individuals from the ZL-ZHŁ-UNFU population formed a separate plot and could also be clearly distinguished from the other populations (EULS and AFES WULS). Similar topologies of genotype groupings from the ZL-ZHŁ-UNFU population in relation to EULS and AFES WULS collections were observed for ISSR and R-ISSR markers, respectively. For ISSR markers, the first two principal components explained 26.1% and 22.1% of the total variation, whereas for R-ISSR 41.7% and 20.9% of total variation were explained, respectively (Fig. 7). In the PCoA plots for RAPD, ISSR, and R-ISSR, greater differences were found between the mean distances of the *Lonicera* genotype pair and the actual distances of the same pairs on the Nei matrix compared to MDS. These differences were 45%, 56%, and 42% higher for RAPD, ISSR, and R-ISSR, respectively (Fig. 7).

## 4 Discussion

R-ISSR has been applied in studies on corn [33], *Arthrocnemum macrostachyum* [34], and rye [36, 37]. It is one of the AAD markers that amplify random DNA sequences, including ISSRs, as well as those “recognized” by different ISSR primers and “shortened” during amplification by complementary RAPDs at a given locus. Thus, the resulting genetic profiles show a series of R-ISSR products in addition to single ISSRs and RAPDs. R-ISSRs are the shortest compared to ISSRs or RAPDs [33, 34, 37].

In the R-ISSR profiles obtained in our study, we identified amplicons with similar electrophoretic mobility to that of the close co-migrating RAPDs and ISSRs, as well as new amplicons (putative heteroamplicons). We found that two R-ISSR products (864 and 468 bp), which were not observed in RAPD or ISSR profiles, like two other products of 713 and 251 bp that migrated close to RAPDs or ISSRs, had heteroamplicon sequences. These findings are in line with the observations presented by Smolik [36, 37]. The significant matches to those heteroamplicon sequences have been identified in CNGB and UniProt, which were *Lonicera japonica* (taxid: 105,884) transcripts from oneKP and MPDB databases and their matches to the Clathrin light domain genes in *Camelia sinensis* or the Integrase catalytic chain in *Vitis vinifera* (UniProt). This supports the fact that R-ISSR markers can be applied in the construction of genetic maps [13, 24, 25] or used as GTMs and FMs [15].

Similar to RAPD, ISSR, AFLP, and RFLP techniques, R-ISSR explores other than the RAPD or ISSR part of the genome, and hence can be used to analyze the range of genetic variability in barcoding or MAS [15, 33, 34]. A total of 258 loci were amplified, of which 83% were polymorphic and genotype-specific (private loci), with the use of 20 primer pairs. Some exceptions were the loci BRA, CZA, WOJ, DEU, C22, C44, and MIN. Each RAPD primer amplified on average 16 loci, of which 87% were polymorphic and genotype-specific (exception ZIE). The length of the amplified products ranged from 3860 to 200 bp. Naugžemys et al. [18] presented similar results for 39 different genotypes of *L. caerulea* using 11 primers which amplified 105 loci with a length of 450–2250 bp. Among the amplified loci, 88 (83.9%) were polymorphic. Naugžemys et al. [9, 19] also analyzed 51 accessions of blue honeysuckle (cultivars and lines) and 12 species of *Lonicera* using 12 primers. The authors amplified 149 and 172 loci with a length of 270–2500 bp, of which 124 (83.2%) and 132 (78.1%) were polymorphic, respectively. Gawroński et al. [38] presented the RAPD results for five cultivars and three clones in their study. Using six primers, the authors only amplified 61 loci with a length of 390–4100 bp, of which 57 (93.3%) were polymorphic. Similar results for *L. caerulea* and *L. japonica* have been presented by other authors [39, 40].

In this study, we amplified 814 loci using 41 ISSRs. Of these loci, 88% were polymorphic, including some private loci. One ISSR primer amplified on average 20 loci. This was the highest number of ISSRs applied to *L. caerulea* compared to the studies of Smolik et al. [20], Lamoureux et al. [46], and Kaczmarska et al. [21], in which 11, 5, and 12 ISSR primers were used, respectively, and 129 and 101 loci were amplified [20, 21]. The percentage of polymorphic loci amplified in these studies was similar and ranged from 83% to 90%, respectively.

The Mantel test demonstrated highly significant correlations (*r*) between the genetic similarity matrices (r_RAPD ISSR_=0.924, r_ISSR R-ISSR_ = 0.938, r_RAPD R-ISSR_ = 0.929) and for all markers (r_RAPD ISSR R-ISSR_ = 0.411). The obtained *r* coefficients indicated the credibility of AAD markers which were used separately to describe the variability and genetic relationships within, for example, *Lonicera* genotypes [41].

Twenty-one genotypes of blue honeysuckle, except L7661, L7662, or L7987 (A-FES WULS), belong to the group of genotypes and their offspring that are currently cultivated and intensively examined in Poland, the Czech Republic, Estonia, Russia, Canada, the US, and China [5, 36, 37, [42], [43], [44], [45]]. The genotypes studied here come from five centers (A-FES, ZL, ZHL, UNFU, and EULS). The genotypes from A-FES WULS are primitive forms native to the Alps (L7661, L7987) and the Far East of Russia (L7662). These were identified as separate clades on the RAPD, ISSR, and R-ISSR dendrograms. The L7661 genotype was found to be the least similar to others.

We studied six genotypes from the EULS collection. These genotypes formed separate clades on the RAPD, ISSR, and R-ISSR dendrograms. The close similarity between these genotypes can probably be attributed to kinship. GOL, SPT, and ZOL were chosen from seeds resulting from open pollination of the selected form Start [19, 47] while ROK, the parent component of the Fialka cultivar [19], from seeds from Kamchatskaya population. The origin of HER and ISK is unknown, and the results of our research suggested they can be closely related to GOL. According to Boyarskikh [47], SPT is the parent component of the Gerda cultivar, and together with GOL and Lazurnaya, it can be the parent component of the Berel variety as well [47]. GOL, SPT, ZOL, Gerda, and Berel have been used in Canadian breeding programs [43]. Due to its unique characteristics, Berel is intensively researched and cultivated in Northeast China [14, 45].

Similar relationships have been described between L7661, L7662, L7987, ZIE, CZAR, WOJ, and ATU (ZHL) using ISSR [20], between GOL, ROK, ZOL, and SPT using RAPD [19], and between ROK and Fialka using AFLP [5].

The analysis of genetic diversity using RAPDs, ISSRs, and R-ISSRs for the ZL, ZHL, and UNFU genotypes showed grouping in clades consistent with their origin. The cultivars BRA, CZA, and ZIE were found to be clustered together. The similarity between ZIE (ZL) and WOJ (ZHL) as indicated by the abovementioned techniques was at the level of 0.83, 0.77, and 0.91, which suggests that these cultivars may have common parental forms. Such cases have already been reported for the other cultivars of L. *caerulea*. For example, for GOL, SPT, and ZOL, the parental form is Start variety, for Tomichka, Vasyuganskaya, and Narymskaya it is Delfin, and for WOL and Chernichka it is Smolinskaya [19, 47].

There are no known pedigrees for the ZHL or ZL cultivars from Polish collections. Using RAPD, ISSR, and R-ISSR, close genetic relationships have been shown between DUE and MIN; JOL, C38, and C22; C44, C46, and DLN/SIN; and DLN, SIN, and WOL. Holubec et al. [5] demonstrated the distinctiveness of DUE from other genotypes from Russian and Chinese collections. It has been found that DLN, SIN, and WOL (UNFU) cultivars are grouped near GOL (EULS), which is consistent with the results of Naugžemys et al. [19]. As these cultivars came from the ZHL collection, we assume that they were used in the breeding programs conducted there.

For RAPD datasets, the maximum logarithmic probability was computed using STRUCTURE (20 runs per K value (K1–10), burn-in period of 100,000 lengths, and 1000,000 MCMC iterations). The ∆*K* value for RAPD was *K* = 2 and then *K* = 4, while for ISSR it is *K* = 2 and *K* = 3, and for R-ISSR, *K* = 3. These additional peaks in Δ*K* suggest the presence of substructures in the RAPD and ISSR datasets (discrete populations) [48]. For analyzing the population structure of *L. caerulea* tetraploid genotypes using dominant markers, the algorithm must be specified which allele (if any) is recessive at each locus [32, 49]. If the ploidy of the tested organism is greater than 2, the estimation of K may be challenging [49]. Based on the RAPD dataset, the STRUCTURE algorithm divided the 24 *Lonicera* genotypes into P1 (A-FES) and P2 (ZL, ZHL, UNFU, EULS) populations, while within P2, three and two subpopulations were identified, respectively (∆*K* = 3, 2) with the following composition: P2.1 (ZL, ZHL), P2.2 (UNFU), and P2.3 (EULS); and P2.1 (ZL, ZHL, UNFU) and P2.2 (EULS). In the case of ISSR, the differences between the estimated values for ∆*K* = 2 and 3 were small. We considered *K* = 3 as a biologically meaningful number [32]. For order, two populations P1.1 (A-FES) and P1.2 (EULS) have been identified within *K* = 2. For R-ISSR, the STRUCTURE algorithm demonstrated the most likely number of populations for *K* = 3 (P1-A-FES; P2-ZL, ZHL, and UNFU; P3-EULS), which was consistent with the data presented for ISSR and RAPD datasets as well as with the divisions generated for the genetic distance determined using the Nei algorithm. By identifying the STRUCTURE divisions of subsequent K (*K* = 4, 5, and 6) for the RAPD and ISSR data, we noticed the trend of subpopulation formation within the ZL+ZHL+UNFU group through the division of ZL+ZHL from UNFU (Fig. S1).

On the MDS and PCoA plots, the *Lonicera* grouping was easily discernible. The MDS algorithm found such 2D configurations, for which the Euclidean distances between the points corresponding to genotypes are the closest to the distances on the genetic distance matrix. The PCoA algorithm also identified and rotated the coordinate system to match the principal component. Thus, the PCoA images show the 2D coordinates of each genotype in the subspace of the two most important principal components. Although the transformed MDS/PCoA matrices showed a significantly positive correlation, in PCoA we observed greater differences between the mean distances of the *Lonicera* genotype pair and the actual distances of the same pairs on the Nei matrix compared to MDS. The MDS images proved to be clearer than the PCoA images.

AMOVA demonstrated that the intra- and interpopulation variability were significant (*P* < 0.001) in all datasets (*F*_ST_RAPD_ = 0.223, *F*_ST_ISSR_ = 0.279, *F*_ST_R-ISSR_ = 0.363), and all the values of *F*_ST_ confirmed a high level of variation [50]. For RAPD, ISSR, and R-ISSR, the differences observed within populations accounted for 78%, 72%, and 64% of the total genetic variations, respectively. This suggests that genetic variations within particular collections are mainly reflected in the differences among genotypes. Naugžemys et al. [19] presented similar results for the genotypes from the EULS collection. The authors noted that genetic differences existed between the cultivar and lineage groups tested with AMOVA. It can be assumed that the differences observed in our work may have resulted from two factors: (1) mutations, genetic drift, and selection [47, 51] and (2) the differences in the composition of the collection *per se* [19]. Among the A-FES WULS genotypes, two were from natural sites in the Alps (Switzerland), and one from Yakutsk (Russia). Although WOJ from the ZL collection was bred by ZHL, it exhibited a close relationship with ZIE (ZL). Based on its phenotypic character, MIN can belong to the ZHL collection. It has large bracts, unlike other ZHL genotypes. It is worth adding that the knowledge of genetic relationships between cultivars can be useful in the selection of pollinators for establishing commercial plantations. Boyarskikh [47] showed that genetically different genotypes, such as those derived from ‘Start’ and ‘Dolphin’, are good pollinators themselves, whereas the related forms are not. If the pedigrees of the cultivars are unknown, pollinator selection should be preceded by performing appropriate tests, including genetic assays.

## 5 Conclusions

Due to its economic importance, the genome of *L. caerulea* L. has been intensively studied in various fields of science. In comparison to RAPD and ISSR, we have demonstrated the usefulness of R-ISSR in the study of genetic diversity and relationships, for the first time for *Lonicera* spp. Similar to R-ISSR markers such as RAPD, ISSR could also be used for core germplasm collection, development of SCAR markers and SSR, genetic map construction, barcoding, protection of variety rights, MAS, and genomic selection. By analyzing the pedigrees of genotypes, we showed that these genotypes are used in the development of germplasm collection in the most important breeding centers in various parts of the world ranging from China to Canada. However, in Poland, genotype collection is carried out differently, as we have demonstrated using the recognized population structure analysis methods, such as MDS or PCoA, and using STRUCTURE software. Using the R language in MDS and PCoA, we have shown how and to what extent the MDS and PCoA algorithms distort the Nei distance matrices after multidimensional data transformation into space (MDS) and 2D subspace (PCoA).

## Author contributions

The authors I.O., M.S. and K.O. received a research grant; M.S., I.O. and P.M. designed the research; L.A., P.B. and I.O. have provided plant material from their collections and taken part in the discussion of the results; M.S., A.B-C. and G.C. performed the amplifications and data analysis with the help of P.M.; J.B. performed the AMOVA analysis; M.S. performed the MDS and PCoA analysis, supervised the research and wrote the manuscript

## Funding

This work was funded by grant N N310 205737 from the Polish Ministry of Science and Higher Education/National Science Center.

## Ethics approval

The studies do not apply to humans or animals. Leaves from plants (genotypes) L7661, L7662, L7987 were obtained for research from the collection and with the permission of the Arboretum in Rogów - Forest Experimental Station WULS (Poland) (PB); from the cultivars: GOL, HER, ISK, ROK, SPT and ZOL from the collection and with the permission of the Estonian University of Life Sciences, Polli Horticultural Research Center, Tartu (Estonia) (LA); cultivars: BRA, CZA, ZIE, WOJ, ATU, DUE, JOL, C22, C38, C44, C46, MIN, DLN, SIN, WOL were collected from mature plants belonged to the collection of the West Pomeranian University of Technology, Szczecin (Poland) (IO) and no special permission is necessary. This collection was created (IO) from plants obtained from their breeders (ZL, ZHŁ) and as a result of bilateral cooperation (UNFU). The investigated plant genotypes are deposited in the above-mentioned collections and there are provided access to this material.

## Consent to participate

Not applicable.

## Consent to publication

Not applicable.

## Declaration of Competing Interest

The authors declare the following financial interests/personal relationships which may be considered as potential competing interests: Krystyna Maria Ostrowska reports financial support was provided by National Science Center Poland.

## Appendix Supplementary materials

Image, application 1

Image, application 2

Image, application 3

Image, application 4
