# Supplementary material for: Fingerprinting, structure, and genetic relationships among selected accessions of blue honeysuckle (Lonicera caerulea L.) from European collections
Source: Biotechnol Rep (Amst). 2022 Mar 23;34:e00721. doi: 10.1016/j.btre.2022.e00721 (PMC9171449; doi:10.1016/j.btre.2022.e00721)
Supplement: Supplementary file 1 [file mmc1.pdf]

Figure S1. Selected columns from the Evanno statistics used to determine true value of K. Each of them contains the graph(s) of the Delta*K* peak(s) according to the results of marker technique used. Data were obtained from Structure harvester. Detailed description was presented below figures

1_RAPD

K	Reps	DeltaK
-	-	-
1	20	-
2	20	4.810
3	20	4.002
4	20	1.232
5	20	1.355
6	20	0.062
7	20	0.146
8	20	0.577
9	20	0.709
10	20	-
-	-	-

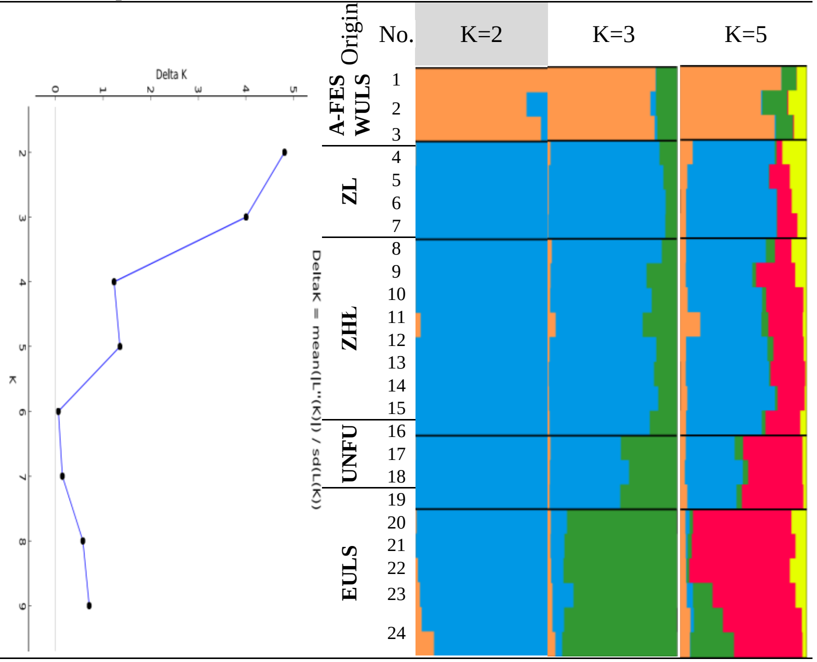

4_RAPD

K	Reps	DeltaK
1	10	-
2	10	3.825
3	10	4.868
4	10	0.622
5	10	0.063
6	10	2.429
7	10	0.709
8	10	1.344
9	10	0.589
10	10	-

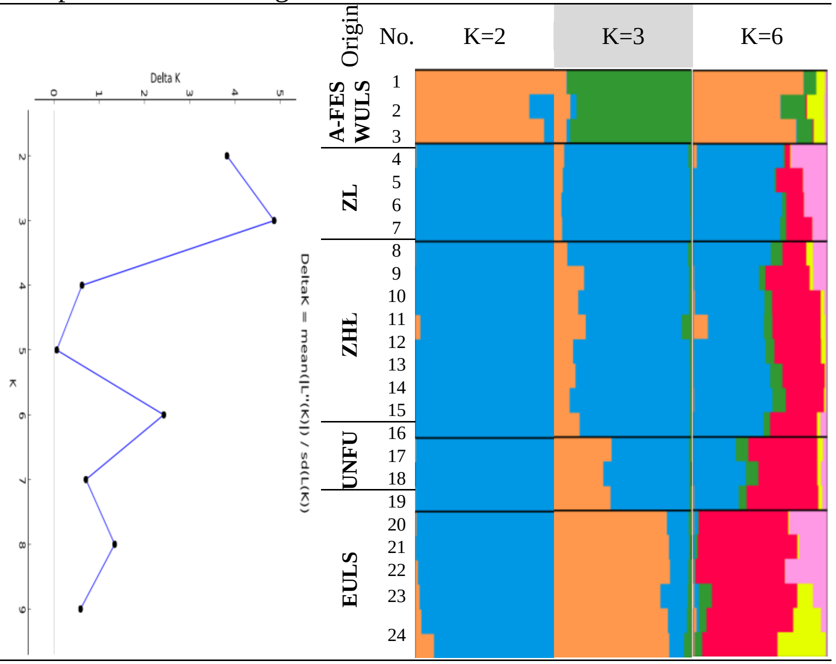

2_ISSR

K	Reps	DeltaK
1	20	-
2	20	10.766
3	20	2.874
4	20	0.861
5	20	0.900
6	20	1.227
7	20	0.466
8	20	0.560
9	20	0.239
10	20	-

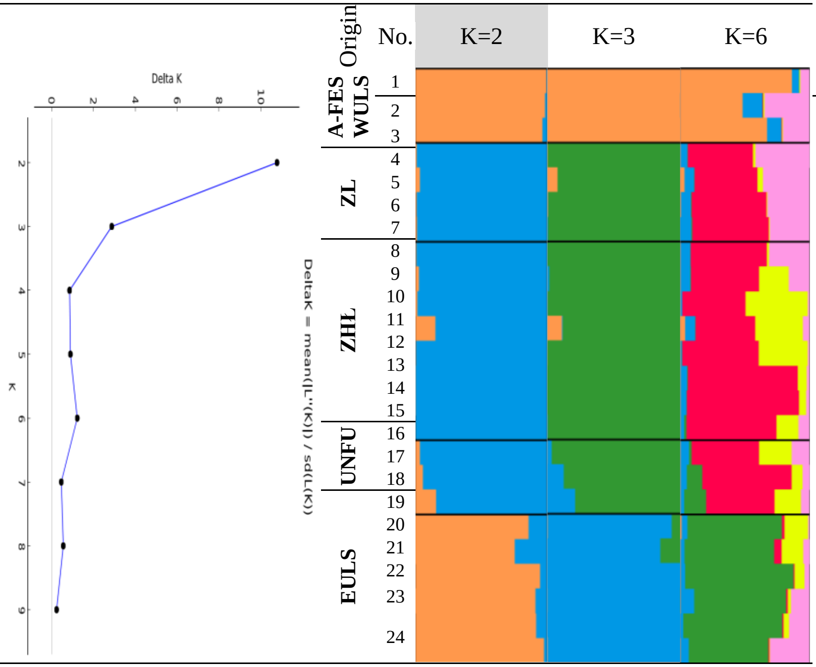

5_ISSR

K	Reps	DeltaK
1	10	-
2	10	8.081
3	10	0.485
4	10	2.032
5	10	1.593
6	10	3.713
7	10	2.853
8	10	0.798
9	10	0.319
10	10	-

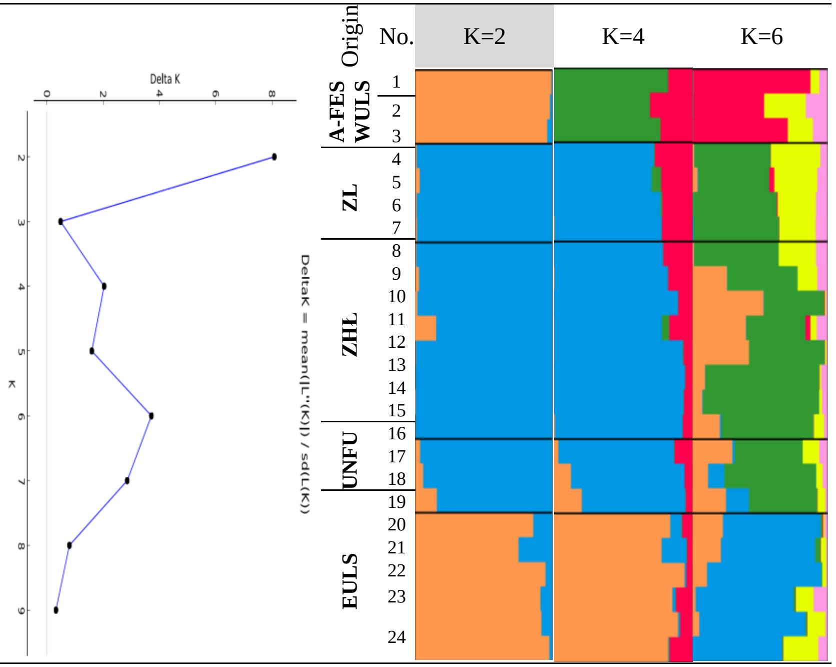

3_R-ISSR

K	Reps	DeltaK
1	20	-
2	20	0.307
3	20	1.918
4	20	1.199
5	20	0.322
6	20	0.678
7	20	0.570
8	20	0.236
9	20	.0.449
10	20	-

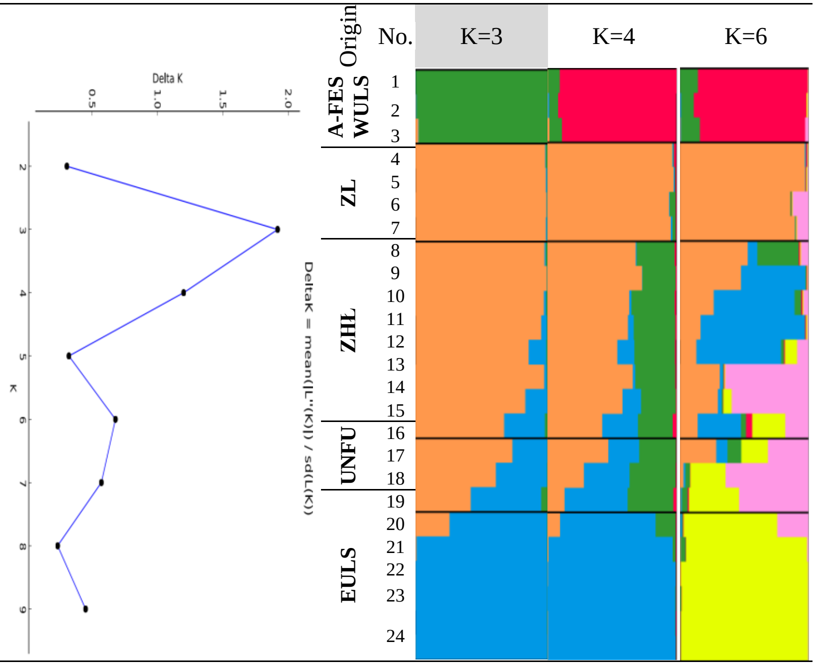

6_R-ISSR

K	Reps	DeltaK
1	10	-
2	10	0.512
3	10	4.420
4	10	1.053
5	10	0.181
6	10	0.357
7	10	0.631
8	10	0.869
9	10	0.348
10	10	-

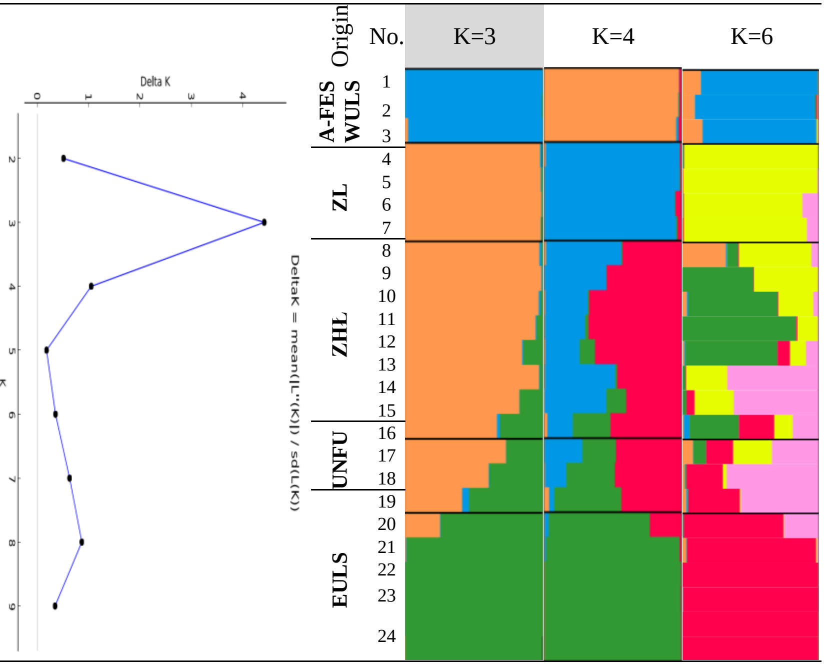

1 - L7661, 2 - L7662, 3 - L7987, 4 - BRA, 5 - CZA, 6 - ZIE, 7 - WOJ, 8 - ATU, 9 - DUE, 10 - JOL, 11 - C22, 12 - C38, 13 - C44, 14 - C46, 15 - DLN, 16 - SIN, 17 - WOL, 18 - MIN, 19 - GOL, 20 - HER, 21 - ISK, 22 - ROK, 23 - SPT, 24 - ZOL

1_RAPD, 2_ISSR, 3_R-ISSR – admixture model and 20 runs, 50,000 burnin, 100,000 MCMC

4_RAPD, 5_ISSR, 6_R-ISSR – admixture model and 10 runs, 50,000 burnin, 100,000 MCMC
