# Supplementary material for: Fingerprinting, structure, and genetic relationships among selected accessions of blue honeysuckle (Lonicera caerulea L.) from European collections
Source: Biotechnol Rep (Amst). 2022 Mar 23;34:e00721. doi: 10.1016/j.btre.2022.e00721 (PMC9171449; doi:10.1016/j.btre.2022.e00721)
Supplement: Supplementary file 2 [file mmc2.pdf]

Supplemental Table S1. Characteristics of RAPD, ISSR and R-ISSRs generated for twenty four genotypes of *Lonicera sp.* from different collections

**RAPD**	Amplicons size range in bp	Loci	L7661	L7662	L7987	BRA	CZA	ZIE	WOJ	ATU	DUE	JOL	Cl2	C38	C44	C46	MIN	DLN	SIN	WOL	GOL	HER	ISK	ROK	SPT	ZOL	**Total number of generated amplicons**	Monomorphic products	Polimorphic products	Genotype-specific products
23	1200-400	20	5	8	6	9	6	9	10	12	8	9	9	8	8	5	5	7	9	10	8	9	7	8	6	4	185	2	16	2
24	3860-220	34	6	7	3	7	9	11	7	8	5	8	6	9	10	8	12	10	14	9	5	3	6	9	10	7	189	0	25	9
30	1790-200	16	4	4	7	7	7	7	6	6	6	5	6	10	5	10	7	7	9	6	6	8	5	9	9	12	168	3	12	1
31	1190-450	11	7	7	7	6	6	6	6	9	7	6	8	6	7	6	7	6	9	8	7	6	8	10	8	8	171	3	6	2
60	1760-340	10	7	7	7	4	8	8	8	7	8	8	8	8	7	6	5	8	8	9	7	7	7	7	8	5	172	4	5	1
63	1530-380	17	6	4	8	4	3	4	5	4	9	6	4	4	8	6	3	5	5	3	7	7	7	10	9	9	140	1	14	2
65	1360-290	24	8	7	5	9	9	9	11	9	12	7	9	12	8	9	9	9	10	9	10	9	11	12	11	10	224	2	14	8
67	1370-270	23	6	7	5	5	7	7	6	7	8	7	7	7	7	6	8	5	7	6	7	7	7	7	7	7	160	1	16	6
73	1240-330	21	7	8	4	5	6	9	6	8	8	5	6	4	6	6	8	6	7	7	8	7	9	9	8	7	164	1	15	5
76	1250-450	12	5	6	6	4	6	7	7	7	7	7	8	5	5	4	5	5	6	5	7	5	5	5	5	3	135	1	9	2
77	1360-250	19	1	9	9	7	6	7	10	11	10	10	10	10	10	10	9	11	9	8	8	8	8	9	8	8	206		12	7
81	1360-350	19	5	5	4	6	6	7	6	8	7	7	6	7	9	8	5	6	9	5	5	8	6	8	6	7	156	2	13	4
82	1830-350	6	5	4	4	5	5	5	4	5	5	5	5	5	4	5	2	4	4	5	4	4	4	4	4	4	105	2	3	1
84	1120-300	13	4	4	6	2	6	4	5	6	6	6	7	7	6	5	6	7	5	4	5	8	3	4	6	6	128	2	8	3
85	2030-550	7	3	4	4	4	4	5	5	4	4	4	4	4	5	6	4	4	5	0	4	4	4	4	4	4	97	0	6	1
208	1470-260	15	8	9	9	8	9	10	10	9	12	10	9	8	10	8	11	9	12	12	9	10	9	10	9	10	230	5	7	3
209	1170-240	9	5	5	6	6	6	6	6	6	3	5	3	5	5	5	6	5	6	6	6	5	4	4	5	6	125	3	4	2
228	1670-340	17	4	5	4	9	11	11	11	9	9	8	9	8	11	11	12	11	12	10	11	9	9	9	10	9	222	2	14	1
230	1350-350	19	8	10	8	6	7	7	6	7	9	8	8	9	7	6	8	8	5	7	10	9	9	7	8	9	186	2	14	3
231	940-380	3	3	3	3	3	3	3	3	3	3	3	3	3	3	3	3	3	3	3	3	3	3	3	3	3	72	3	0	0
232	1060-540	7	3	5	4	3	3	3	3	3	3	3	3	3	3	3	3	3	3	4	5	4	4	5	3	4	83	1	4	2
234	1130-540	6	4	4	4	4	4	4	4	4	4	4	4	4	4	4	4	4	4	4	4	4	4	4	4	4	96	3	2	1
238	1330-470	11	6	3	4	3	3	5	5	5	2	4	3	4	6	4	5	2	2	4	0	4	4	4	3	3	88	0	8	3
244	1340-310	18	5	5	7	7	7	8	7	8	6	6	7	7	7	7	6	9	6	6	11	9	10	8	7	8	174	3	9	6
245	1510-330	18	10	8	7	7	6	8	7	8	7	9	10	9	9	10	7	8	9	8	8	8	8	8	9	7	195	5	11	2
248	1400-320	22	6	9	3	8	6	6	5	8	8	7	7	7	6	6	5	8	6	5	6	5	3	6	6	5	147	1	17	4
251	1970-300	17	5	5	5	4	5	4	4	3	4	5	4	0	4	4	4	6	3	6	5	5	5	5	5	5	105	0	10	7
277	1090-370	9	8	7	6	5	5	4	5	4	4	5	5	5	5	5	5	6	5	5	5	5	5	5	5	5	124	4	2	3

280	1180-340	17	6	7	5	1	6	6	6	4	6	6	4	4	7	8	4	7	9	9	8	7	8	5	5	6	144	0	17	0
282	1340-300	16	7	8	9	6	8	0	9	10	8	10	10	10	10	9	8	11	9	10	10	9	9	8	8	10	206	0	16	0
285	1430-200	20	8	12	9	7	9	6	7	12	8	10	11	10	11	9	9	11	9	9	10	8	10	8	8	7	218	4	15	1
287	1160-310	11	7	7	6	4	4	4	4	4	6	5	5	5	5	5	5	5	5	4	5	5	5	4	4	7	120	3	7	1
290	1600-290	18	12	12	11	9	12	8	10	12	12	13	13	11	11	11	12	11	12	11	12	11	11	11	10	11	269	7	9	2
292	1570-280	27	6	10	6	5	4	7	7	10	9	9	2	11	7	8	7	7	8	7	7	5	5	7	8	7	169	0	18	9
296	1450-270	20	7	7	8	6	9	8	9	10	8	11	9	10	8	7	8	9	9	11	10	9	9	11	8	8	209	3	9	8
299	1240-310	15	2	3	3	5	4	4	3	8	5	5	4	5	3	5	6	5	4	7	3	3	3	4	6	2	102	2	7	6
300	1310-230	15	4	2	3	3	3	2	2	3	3	4	1	1	3	1	1	4	4	1	5	4	5	4	3	7	73	0	10	5
303	2620-300	15	6	9	6	5	4	6	5	4	5	5	7	5	5	7	6	9	5	5	8	7	7	9	8	6	149	4	7	4
305	1830-320	19	9	9	9	9	12	6	12	9	10	9	10	10	12	10	10	9	12	8	9	10	13	11	11	5	234	4	13	2
312	1510-300	19	7	13	11	10	11	10	10	9	10	9	10	9	9	8	9	10	9	9	10	9	10	10	8	7	227	3	13	3
313	1790-350	27	5	11	10	9	10	8	8	8	15	10	9	6	9	4	13	7	11	8	12	9	10	10	11	11	224	1	22	4
318	1700-410	14	4	6	5	6	10	12	10	11	10	9	7	9	11	10	10	9	11	8	9	8	9	9	9	9	211	4	10	0
322	1680-360	16	3	5	5	8	7	6	7	8	10	8	3	8	8	5	7	8	9	9	9	9	8	9	4	6	169	1	14	1
	**Total**	692	247	290	261	250	282	277	287	310	309	300	283	292	304	283	289	304	318	290	308	293	296	313	297	288	6971	92	463	137
	**Mean**	43	6	7	6	6	7	6	7	7	7	7	7	7	7	7	7	7	7	7	7	7	7	7	7	7	162	2	11	3

**ISSR**	Amplicons size range in bp	Loci	L7661	L7662	L7987	BRA	CZA	ZIE	WOJ	ATU	DUE	JOL	Cl2	C38	C44	C46	MIN	DLN	SIN	WOL	GOL	HER	ISK	ROK	SPT	ZOL	**Total number of generated amplicons**	Monomorphic products	Polimorphic products	Genotype-specific.products
817	1950-440	13	7	7	8	7	7	10	10	9	9	9	9	10	10	9	10	7	10	11	8	10	9	10	8	9	213	2	11	0
818	1910-410	31	12	10	10	5	8	9	10	2	10	3	13	10	8	3	10	8	9	5	12	9	5	11	3	11	196	0	27	4
826	2380-320	17	7	7	8	9	10	12	8	12	12	11	13	9	11	9	9	13	12	12	11	9	12	13	13	14	256	2	15	0
829	1500-320	21	6	6	2	9	9	5	6	6	6	7	6	8	7	7	9	7	8	8	10	9	10	5	10	11	177	0	18	3
830	1780-400	18	6	7	10	11	12	7	8	11	12	12	11	11	10	12	11	10	9	9	7	7	7	7	9	6	222	2	16	0
840	1510-220	18	10	12	11	10	14	10	12	12	14	11	12	12	15	14	13	14	13	11	11	9	10	14	11	4	279	3	14	1
845	2010-330	26	11	14	8	8	12	7	7	8	11	9	10	8	8	7	8	11	6	11	10	11	6	8	9	9	217	1	22	3
846	2790-370	25	6	6	5	5	5	4	6	7	11	8	7	5	7	6	7	10	6	8	16	7	12	8	9	10	181	1	21	3
853	1990-500	16	7	9	9	8	10	9	8	10	9	8	8	9	8	9	7	9	9	8	10	8	8	8	9	10	207	4	9	3
857	2440-390	22	7	7	8	9	4	10	12	7	8	9	11	10	9	8	8	5	8	9	10	9	7	11	9	7	202	1	18	3
858	2130-420	13	7	8	8	7	8	8	8	8	9	9	8	9	8	8	9	9	8	9	8	8	7	8	8	8	195	6	5	2
859	2250-250	24	7	9	6	2	7	4	10	4	9	11	9	9	10	7	6	9	10	12	8	10	11	7	7	8	192	1	21	2

861	1390-600	9	5	5	5	5	8	6	7	5	8	8	8	8	8	8	8	8	8	8	8	8	8	8	8	8	174	4	4	1
866	2580-400	22	10	9	10	6	9	10	9	10	11	9	10	11	10	8	9	6	10	10	10	7	8	7	5	7	211	0	20	3
874	2060-390	16	6	5	9	8	9	9	7	10	9	10	10	7	8	9	8	10	8	8	10	7	11	11	9	5	203	1	14	1
878	2640-590	19	10	8	6	11	4	11	10	10	10	10	10	13	11	12	10	11	12	10	12	11	10	10	10	6	238	0	18	1
881	2430-230	26	15	13	9	13	12	14	11	12	15	14	8	8	15	15	13	13	13	12	18	13	15	9	12	12	304	3	22	1
808*	2090-230	35	9	8	14	11	12	13	9	9	12	12	13	14	10	15	12	14	12	12	10	10	11	12	10	3	267	1	28	6
819*	1550-280	15	6	7	7	4	3	5	5	6	6	5	6	6	5	6	6	5	7	6	8	7	6	8	9	7	146	1	13	1
830*	1240-200	17	8	8	5	6	7	6	6	10	4	5	7	6	6	9	7	7	7	8	6	7	6	7	5	8	161	1	12	4
831*	2200-480	13	7	6	7	4	4	6	6	7	4	5	4	5	6	6	8	8	5	6	6	5	7	6	5	7	140	2	11	0
839*	1980-230	14	8	8	8	6	8	7	8	9	11	9	9	10	10	7	9	12	12	11	12	11	10	10	8	7	220	4	10	0
840*	1560-350	13	10	10	9	9	10	8	8	8	10	10	10	10	9	9	9	10	10	11	12	12	12	12	11	11	240	6	7	0
841*	1420-270	12	5	6	8	8	8	10	10	8	8	10	11	10	9	9	10	10	9	9	9	9	8	8	6	6	204	2	10	0
843*	1540-260	16	6	7	7	7	8	7	7	9	8	8	7	8	7	7	9	8	8	9	11	10	10	14	11	10	203	5	9	2
844*	980-330	11	6	8	5	4	4	5	5	4	6	5	7	5	5	5	5	5	5	5	9	8	7	9	9	8	144	4	6	1
845*	1770-320	15	6	7	7	7	10	6	10	6	10	9	9	9	9	7	9	9	10	9	12	11	11	10	11	8	212	5	9	1
846*	1120-400	12	5	6	5	3	3	3	3	3	4	5	5	5	4	4	4	5	4	5	7	7	8	6	5	6	115	2	8	2
849*	2050-320	20	7	7	7	5	7	7	6	8	7	5	7	5	6	7	6	6	10	11	8	8	8	8	8	8	172	2	14	4
850*	1720-310	26	5	6	8	5	5	4	6	4	8	5	5	2	7	3	5	5	6	4	8	7	6	9	7	8	138	0	20	6
854*	1960-400	18	7	8	5	7	9	7	9	9	9	11	11	11	9	9	9	8	9	8	8	8	9	10	8	9	207	3	10	5
857*	2280-310	26	9	9	11	10	9	12	10	9	17	11	10	14	13	14	12	9	14	14	12	10	11	11	10	9	270	3	22	1
862*	1740-370	16	6	11	8	9	7	10	10	9	8	9	7	9	10	10	7	7	11	9	11	11	11	9	13	10	222	1	13	2
st7	2780-280	36	10	9	5	10	8	10	11	11	9	10	9	11	7	12	14	8	13	12	7	11	3	14	6	8	228	0	30	6
st8	2460-270	34	9	10	12	7	10	15	6	11	12	13	8	9	12	11	19	14	11	14	10	11	8	14	14	8	268	0	29	5
st9	2000-260	25	8	8	6	8	11	8	10	9	11	14	13	10	4	10	8	12	13	8	11	12	11	11	11	11	238	0	22	3
st10	1940-550	16	6	10	6	8	9	7	7	7	7	11	11	11	7	7	10	9	9	11	10	9	9	10	10	7	208	2	13	1
st11	1840-260	23	7	8	7	12	12	10	12	13	13	13	11	13	14	15	13	10	13	12	10	12	12	10	9	8	269	3	16	4
st12	1350-300	29	7	6	8	5	7	5	6	4	10	5	13	3	9	4	2	5	5	4	9	9	10	7	8	6	157	0	24	5
st17	1430-290	17	6	8	7	6	5	8	8	6	6	9	10	8	8	8	7	9	8	9	6	9	6	9	8	6	180	3	11	2
st20	2290-400	19	8	6	12	7	7	8	8	7	11	10	9	8	7	8	9	8	9	7	9	9	7	11	9	5	199	0	17	2
	Total	814	310	329	316	301	331	332	335	329	384	367	375	359	356	353	364	363	379	375	400	375	363	390	360	329	8475	81	639	94
	Mean	20	8	8	8	7	8	8	8	8	9	9	9	9	9	9	9	9	9	9	10	9	9	10	9	8	207	2	16	2

Oligos and their sequence signed in the manuscript as *Pr* and with asterix (*) has been taken from other publications. Sequences are presented below (5’-3’)

808*[AG]$_8$C 819* 831* 830* 839* 840* 841* 843* 844* 845* 846*
849* 850* 854*[TG]$_8$C 857*[AC]$_8$GGTC 862*[AC]$_8$YGG Pr7 [CA]$_7$GG Pr8 [CA]$_7$CG Pr9 [GA]$_7$GT Pr10 [GT]$_7$GG Pr11 [GA]$_7$CC Pr12 [GT]$_7$CC
Pr17 [AG]$_8$G Pr20 [CA]$_8$G

R-ISSR	Amplicons size range in bp	Loci	L7661	L7662	L7987	BRA	CZA	ZIE	WOJ	ATU	DUE	JOL	Cl2	C38	C44	C46	MIN	DLN	SIN	WOL	GOL	HER	ISK	ROK	SPT	ZOL	**Total number of generated amplicons**	Monomorphic products	Polimorphic products	Genotype-specific,products
81+835	1710-380	5	3	3	3	4	4	4	4	4	4	4	4	4	4	4	4	5	4	4	5	4	4	4	4	4	95	3	2	0
81+872	1460-160	12	6	6	8	7	7	8	7	5	4	4	8	8	10	9	7	10	10	9	7	5	6	6	5	4	166	2	10	0
86+876	2900-250	13	6	7	7	8	8	9	8	10	9	7	8	8	7	7	7	8	6	7	7	6	5	6	7	8	176	6	6	1
211+835	1770-280	17	6	4	7	7	8	9	9	10	11	9	11	10	10	10	12	10	10	8	8	7	6	7	7	6	202	1	10	6
211+876	1910-150	20	8	11	9	10	10	10	10	11	10	12	8	11	10	11	9	10	11	9	9	11	11	8	10	10	239	1	11	8
226+835	1860-270	12	5	5	6	8	8	7	8	8	10	9	10	9	8	7	9	6	8	7	7	7	7	7	7	7	180	2	9	1
226+876	1620-150	12	9	6	5	7	8	8	7	9	7	5	6	3	6	5	6	7	6	7	6	6	7	8	7	6	157	1	10	1
245+872	2900-1000	10	5	6	6	6	7	8	8	8	8	8	9	8	9	9	8	9	8	8	8	8	8	8	8	8	186	5	5	0
248+876	1300-280	9	3	3	4	3	3	4	3	5	2	2	2	2	4	2	3	3	2	3	5	3	2	3	3	3	72	2	6	1
251+835	1790-330	13	4	3	6	5	6	4	6	9	6	8	6	8	8	9	9	8	6	6	6	7	5	7	7	7	156	1	10	2
251+876	1220-150	8	4	2	1	5	5	5	5	3	3	5	3	3	6	6	3	6	3	3	5	4	3	3	3	3	92	1	5	2
615+811	1460-110	21	8	11	9	10	10	10	10	11	10	12	8	11	10	11	9	10	11	9	9	11	11	8	10	10	239	1	12	8
615+835	1700-210	14	7	8	7	8	8	8	8	10	9	9	10	8	10	10	10	11	11	11	10	9	9	8	7	7	213	4	9	1
615+844	2410-220	12	5	4	4	3	3	3	3	2	4	4	3	4	4	4	4	4	6	4	4	6	4	4	5	4	95	3	7	2
615+872	1440-220	10	6	4	5	7	7	5	7	7	8	8	8	8	4	5	6	8	7	7	7	6	6	6	6	6	154	2	8	0
615+876	1470-290	14	11	7	6	7	7	6	7	8	6	8	7	7	9	8	7	7	6	7	7	6	7	7	6	6	170	4	8	2
693+811	1220-280	7	3	4	3	4	4	5	4	5	4	5	5	5	5	5	5	6	5	4	4	5	4	6	6	4	110	2	5	0
646+835	1860-270	17	5	4	7	6	6	5	4	8	4	6	3	6	4	2	11	6	5	7	4	5	2	5	4	0	119	0	12	5
693+835	2170-190	16	6	3	6	8	11	10	8	8	10	10	8	8	9	11	10	11	9	9	10	8	8	8	8	8	205	2	12	2
693+876	2050-210	16	4	6	6	7	8	11	8	7	6	8	8	8	8	10	8	8	6	5	6	7	6	6	4	3	164	1	12	3
	**Total**	**258**	114	107	115	130	138	139	134	148	135	143	135	139	145	145	147	153	140	134	134	131	121	125	124	114	**3190**	44	169	45
	**Mean**	13	6	5	6	7	7	7	7	7	7	7	7	7	7	7	7	8	7	7	7	7	6	6	6	6	160	2	8	2
