# Supplementary material for: Fingerprinting, structure, and genetic relationships among selected accessions of blue honeysuckle (Lonicera caerulea L.) from European collections
Source: Biotechnol Rep (Amst). 2022 Mar 23;34:e00721. doi: 10.1016/j.btre.2022.e00721 (PMC9171449; doi:10.1016/j.btre.2022.e00721)
Supplement: Supplementary file 3 [file mmc3.pdf]

Table S2. Primers and accession-specific (private) amplicons

Accession	Primer(s) and accession-specific amplicons generated in bp		
	RAPD	ISSR	R-ISSR
*L*7661	$24_{[1330; 400]}$ $31_{[710]}$ $65_{[810]}$ $82_{[510]}$ $208_{[590]}$ $234_{[1130]}$ $238_{[1170; 840]}$ $244_{[1300]}$ $245_{[820; 750]}$ $248_{[860]}$ $277_{[720; 570]}$ $290_{[1200; 1100]}$ $296_{[730]}$	$818_{[770]}$ $829_{[320]}$ $874_{[390]}$ $849*_{[2050]}$ $862*_{[880]}$ $Pr9_{[330]}$	$211+835_{[1090, 520]}$ $211+876_{[150]}$ $615+811_{[110]}$ $615+876_{[990, 750]}$ $646+835_{[520]}$ $693+835_{[1140]}$ $693+876_{[290]}$
*L*7662	$24_{[2350]}$ $67_{[680; 640]}$ $73_{[1060]}$ $77_{[1070; 730]}$ $81_{[1170]}$ $230_{[1350]}$ $232_{[750]}$ $248_{[1400]}$ $277_{[650]}$ $285_{[1430]}$ $292_{[1080; 680; 300]}$ $303_{[2120; 700]}$ $312_{[1420; 1350]}$ $313_{[440]}$	$829_{[1500]}$ $845_{[2010, 580, 440]}$ $853_{[1200]}$ $858_{[1500]}$ $861_{[740]}$ $866_{[860]}$ $819*_{[1300]}$ $830*_{[1180, 760]}$ $844*_{[770]}$ $849*_{[1130]}$ $850*_{[810]}$ $854*_{[1610]}$ $Pr12_{[360]}$	$211+876_{[1910]}$ $226+876_{[320]}$ $615+811_{[1460]}$
*L*7987	$73_{[570]}$ $76_{[720]}$ $77_{[1250; 980]}$ $84_{[300]}$ $209_{[960; 390]}$ $232_{[930]}$ $244_{[1020; 570]}$ $251_{[460]}$ $296_{[830]}$ $312_{[1510]}$ $313_{[830]}$	$853_{[880]}$ $866_{[740]}$ $849*_{[1490]}$ $Pr7_{[260]}$ $Pr8_{[2210, 280]}$ $Pr9_{[1120]}$ $Pr11_{[560]}$ $Pr17_{[1430]}$ $Pr20_{[1320]}$	$211+835_{[940]}$ $211+876_{[550, 300]}$ $251+835_{[850]}$ $615+811_{[470, 210]}$ $646+835_{[390]}$
BRA	$73_{[790]}$ $303_{[300]}$ $305_{[620]}$	$818_{[930]}$ $830*_{[270]}$ $850*_{[500]}$ $Pr8_{[270]}$	-
CZA	$251_{[1260]}$	$808*_{[1440, 1320, 680]}$ $Pr12_{[320]}$	-
ZIE	-	$853_{[720]}$ $808*_{[230]}$	$646+835_{[510]}$ $693+876_{[1680]}$
WOJ	$65_{[520]}$ $305_{[1150]}$	$857_{[2320, 790]}$ $850*_{[1040]}$ $Pr12_{[1140]}$	-
ATU	$65_{[950; 380]}$ $67_{[970]}$ $77_{[400]}$ $299_{[830; 760; 540]}$	$866_{[400]}$ $808*_{[400]}$ $Pr7_{[560]}$ $Pr11_{[500]}$ $830*_{[450]}$ $849*_{[470]}$	$86+876_{[1300]}$ $211+876_{[900]}$ $251+835_{[1190]}$ $615+811_{[840]}$ $615+835_{[430]}$
DUE	$208_{[1070]}$	$846_{[370]}$ $881_{[2430]}$ $845*_{[1540]}$ $857*_{[2020]}$	
JOL	$24_{[1140]}$ $65_{[530]}$	$859_{[430, 340]}$ $Pr9_{[1770]}$ $Pr11_{[1230]}$	$251+876_{[820]}$
C22	$23_{[980]}$ $67_{[1220]}$ $76_{[700]}$ $81_{[820]}$ $251_{[690]}$ $292_{[830; 280]}$ $313_{[640]}$	$857_{[1320]}$ $878_{[2640]}$ $Pr10_{[1880]}$	
C38	$24_{[790]}$ $60_{[570]}$ $81_{[350]}$ $84_{[630]}$ $296_{[1080]}$	$Pr11_{[640]}$	$646+835_{[450]}$ $693+835_{[410]}$
C44	$24_{[420]}$ $238_{[1100]}$	$Pr12_{[1420]}$	-
C46	$81_{[430]}$ $85_{[550]}$ $299_{[390]}$	$830*_{[830]}$ $Pr7_{[2450, 1250]}$ $Pr8_{[1300]}$ $Pr20_{[1060]}$	$211+876_{[600]}$ $615+811_{[550]}$
MIN	$67_{[370]}$ $296_{[490]}$ $313_{[1090]}$	$843*_{[490]}$ $850*_{[370]}$	-
DLN	$77_{[550]}$ $251_{[530]}$	$858_{[1210]}$ $850*_{[470]}$ $Pr12_{[1670]}$	$211+835_{[1330, 1200]}$ $646+835_{[1400]}$
SIN	$23_{[870]}$ $65_{[410]}$ $300_{[720; 560]}$	$Pr7_{[2780]}$	$693+876_{[800]}$
WOL	$251_{[1970]}$ $296_{[720]}$ $299_{[1240]}$	$846_{[570]}$	$211+876_{[1130]}$ $226+835_{[480]}$ $615+811_{[1090]}$
GOL	$244_{[930]}$	$Pr7_{[280]}$	$248+876_{[280]}$
HER	$30_{[1790]}$ $67_{[1370]}$ $84_{[1120]}$ $230_{[460]}$ $248_{[1160]}$ $303_{[480]}$	$Pr8_{[720]}$	$251+876_{[350]}$ $615+844_{[350]}$
ISK	$65_{[610]}$ $73_{[920]}$	$846*_{[1070]}$ $850*_{[1420]}$	-
ROK	$31_{[870]}$ $63_{[560]}$ $73_{[900]}$ $77_{[570]}$ $244_{[310]}$ $248_{[1300]}$ $251_{[920; 430]}$ $292_{[1090]}$ $296_{[270]}$	$818_{[670]}$ $840_{[700]}$ $843*_{[260]}$ $854*_{[1960]}$ $Pr17_{[300]}$	$211+835_{[340]}$
SPT	$24_{[550]}$ $65_{[1360]}$ $230_{[770]}$ $292_{[390;330]}$ $296_{[760]}$ $299_{[310]}$	$862*_{[1740]}$	$615+844_{[2410]}$
ZOL	$24_{[1440; 800]}$ $63_{[580]}$ $208_{[1030]}$ $228_{[340]}$ $244_{[420]}$ $287_{[400]}$ $292_{[360]}$ $296_{[1230]}$ $300_{[1310; 300; 230]}$ $322_{[850]}$	$818_{[590]}$ $829_{[340]}$ $846_{[430]}$ $808*_{[810]}$ $846*_{[400]}$ $854*_{[590, 510, 450]}$	$211+876_{[560]}$ $615+811_{[490]}$
