# Supplementary material for: Fingerprinting, structure, and genetic relationships among selected accessions of blue honeysuckle (Lonicera caerulea L.) from European collections
Source: Biotechnol Rep (Amst). 2022 Mar 23;34:e00721. doi: 10.1016/j.btre.2022.e00721 (PMC9171449; doi:10.1016/j.btre.2022.e00721)
Supplement: Supplementary file 4 [file mmc4.pdf]

Supplemental Table S3. Matrices of genetic similarity presented for *Lonicera* genotypes according to RAPD, ISSR and R-ISSR results, respectively according to Jaccard’s and Nei’s coefficients

RAPD

Jaccard

**Nei**	L7661	L7662	L7987	BRA	CZA	ZIE	WOJ	ATU	DUE	JOL	Cl2	C38	C44	C46	MIN	DLN	SIN	WOL	GOL	HER	ISK	ROK	SPT	ZOL
*L*7661	**1.00**	0.25	0.25	0.25	0.26	0.24	0.26	0.27	0.26	0.27	0.29	0.28	0.27	0.25	0.27	0.25	0.25	0.23	0.28	0.27	0.29	0.29	0.27	0.27
*L*7662	0.40	**1.00**	0.57	0.49	0.51	0.48	0.49	0.48	0.51	0.53	0.52	0.50	0.50	0.50	0.50	0.48	0.49	0.47	0.53	0.51	0.51	0.51	0.51	0.46
*L*7987	0.40	0.73	**1.00**	0.49	0.53	0.47	0.50	0.47	0.48	0.51	0.53	0.50	0.50	0.49	0.47	0.48	0.49	0.46	0.49	0.49	0.49	0.50	0.49	0.47
BRA	0.40	0.66	0.65	**1.00**	0.69	0.58	0.61	0.56	0.59	0.56	0.54	0.56	0.55	0.56	0.52	0.55	0.56	0.56	0.55	0.54	0.53	0.54	0.53	0.49
CZA	0.42	0.67	0.69	0.82	**1.00**	0.66	0.73	0.65	0.68	0.65	0.64	0.63	0.65	0.61	0.62	0.63	0.62	0.63	0.63	0.62	0.60	0.62	0.60	0.54
ZIE	0.39	0.65	0.64	0.74	0.79	**1.00**	0.71	0.65	0.61	0.61	0.58	0.59	0.64	0.63	0.56	0.60	0.60	0.59	0.59	0.58	0.57	0.59	0.57	0.51
WOJ	0.41	0.66	0.66	0.76	0.84	0.83	**1.00**	0.67	0.62	0.66	0.62	0.62	0.73	0.67	0.63	0.68	0.64	0.60	0.62	0.63	0.59	0.60	0.59	0.53
ATU	0.43	0.65	0.64	0.72	0.79	0.79	0.80	**1.00**	0.66	0.63	0.60	0.61	0.62	0.59	0.58	0.60	0.59	0.61	0.58	0.57	0.55	0.58	0.58	0.50
DUE	0.41	0.67	0.65	0.74	0.81	0.76	0.77	0.79	**1.00**	0.68	0.65	0.62	0.65	0.61	0.62	0.62	0.59	0.69	0.63	0.62	0.63	0.61	0.61	0.55
JOL	0.42	0.69	0.67	0.72	0.79	0.76	0.80	0.77	0.81	**1.00**	0.68	0.77	0.68	0.61	0.65	0.63	0.64	0.62	0.62	0.62	0.59	0.60	0.60	0.53
C22	0.44	0.68	0.69	0.70	0.78	0.74	0.77	0.75	0.79	0.81	**1.00**	0.68	0.65	0.64	0.61	0.61	0.63	0.62	0.63	0.60	0.59	0.60	0.62	0.53
C38	0.43	0.67	0.67	0.72	0.77	0.75	0.77	0.76	0.76	0.87	0.81	**1.00**	0.66	0.64	0.64	0.61	0.63	0.62	0.60	0.59	0.57	0.59	0.60	0.55
C44	0.42	0.67	0.67	0.71	0.79	0.78	0.85	0.77	0.79	0.81	0.79	0.80	**1.00**	0.77	0.68	0.74	0.63	0.64	0.65	0.63	0.61	0.62	0.58	0.56
C46	0.40	0.66	0.65	0.72	0.75	0.78	0.80	0.74	0.76	0.75	0.78	0.78	0.87	**1.00**	0.64	0.66	0.61	0.62	0.59	0.59	0.57	0.59	0.58	0.53
MIN	0.43	0.66	0.64	0.68	0.76	0.72	0.77	0.74	0.76	0.78	0.76	0.78	0.81	0.78	**1.00**	0.62	0.62	0.65	0.65	0.65	0.62	0.62	0.63	0.56
DLN	0.41	0.65	0.65	0.71	0.77	0.75	0.81	0.75	0.77	0.77	0.76	0.76	0.85	0.80	0.77	**1.00**	0.73	0.64	0.67	0.65	0.62	0.65	0.61	0.56
SIN	0.40	0.66	0.66	0.71	0.77	0.75	0.78	0.74	0.74	0.78	0.77	0.77	0.77	0.75	0.77	0.85	**1.00**	0.63	0.66	0.65	0.61	0.62	0.63	0.56
WOL	0.38	0.64	0.63	0.72	0.77	0.75	0.75	0.76	0.82	0.76	0.76	0.76	0.78	0.77	0.79	0.78	0.77	**1.00**	0.60	0.57	0.56	0.60	0.59	0.53
GOL	0.43	0.69	0.66	0.71	0.78	0.74	0.77	0.73	0.77	0.77	0.77	0.75	0.78	0.74	0.78	0.80	0.80	0.75	**1.00**	0.73	0.79	0.67	0.65	0.60
HER	0.43	0.68	0.66	0.70	0.76	0.74	0.77	0.73	0.77	0.77	0.75	0.74	0.77	0.74	0.79	0.79	0.79	0.73	0.84	**1.00**	0.76	0.68	0.65	0.58
ISK	0.45	0.68	0.66	0.69	0.75	0.73	0.74	0.71	0.77	0.74	0.74	0.73	0.76	0.73	0.77	0.76	0.76	0.72	0.88	0.86	**1.00**	0.67	0.64	0.57
ROK	0.45	0.68	0.67	0.70	0.76	0.75	0.75	0.73	0.76	0.75	0.75	0.74	0.76	0.74	0.76	0.79	0.77	0.75	0.80	0.81	0.80	**1.00**	0.69	0.59
SPT	0.43	0.67	0.66	0.69	0.75	0.72	0.74	0.73	0.76	0.75	0.77	0.75	0.74	0.73	0.77	0.76	0.77	0.74	0.79	0.79	0.78	0.82	**1.00**	0.66
ZOL	0.43	0.63	0.64	0.65	0.70	0.68	0.69	0.66	0.71	0.69	0.69	0.71	0.72	0.69	0.72	0.72	0.72	0.70	0.75	0.74	0.72	0.75	0.80	**1.00**

ISSR

Jaccard

**Nei**	L7661	L7662	L7987	BRA	CZA	ZIE	WOJ	ATU	DUE	JOL	Cl2	C38	C44	C46	MIN	DLN	SIN	WOL	GOL	HER	ISK	ROK	SPT	ZOL
*L*7661	**1.00**	0.21	0.21	0.24	0.27	0.28	0.29	0.27	0.31	0.27	0.27	0.29	0.31	0.28	0.30	0.30	0.29	0.29	0.27	0.29	0.25	0.26	0.27	0.24
*L*7662	0.34	**1.00**	0.48	0.43	0.43	0.44	0.41	0.41	0.42	0.42	0.41	0.40	0.42	0.41	0.41	0.41	0.44	0.41	0.42	0.42	0.41	0.42	0.40	0.39
*L*7987	0.35	0.64	**1.00**	0.42	0.44	0.40	0.40	0.39	0.42	0.40	0.41	0.41	0.40	0.41	0.40	0.41	0.41	0.40	0.39	0.39	0.39	0.40	0.37	0.34
BRA	0.39	0.60	0.59	**1.00**	0.59	0.54	0.54	0.55	0.55	0.53	0.46	0.52	0.52	0.54	0.56	0.51	0.51	0.51	0.42	0.46	0.44	0.45	0.47	0.42
CZA	0.43	0.60	0.61	0.74	**1.00**	0.54	0.59	0.57	0.59	0.54	0.53	0.53	0.53	0.53	0.56	0.55	0.56	0.54	0.48	0.49	0.47	0.48	0.48	0.42
ZIE	0.44	0.61	0.57	0.70	0.70	**1.00**	0.63	0.59	0.52	0.52	0.51	0.54	0.57	0.60	0.54	0.52	0.57	0.56	0.48	0.49	0.46	0.49	0.46	0.45
WOJ	0.45	0.58	0.57	0.70	0.74	0.77	**1.00**	0.55	0.53	0.53	0.53	0.56	0.62	0.59	0.54	0.53	0.62	0.52	0.47	0.50	0.49	0.52	0.49	0.45
ATU	0.43	0.58	0.56	0.71	0.72	0.74	0.71	**1.00**	0.58	0.58	0.51	0.55	0.55	0.58	0.57	0.56	0.56	0.54	0.47	0.49	0.45	0.49	0.48	0.44
DUE	0.48	0.59	0.59	0.71	0.74	0.69	0.70	0.73	**1.00**	0.63	0.58	0.60	0.59	0.58	0.65	0.60	0.59	0.57	0.53	0.52	0.51	0.50	0.50	0.44
JOL	0.43	0.59	0.57	0.69	0.70	0.68	0.69	0.73	0.77	**1.00**	0.65	0.72	0.59	0.58	0.61	0.60	0.58	0.61	0.54	0.55	0.51	0.49	0.51	0.43
C22	0.43	0.58	0.58	0.63	0.69	0.67	0.69	0.68	0.74	0.78	**1.00**	0.63	0.58	0.56	0.57	0.56	0.58	0.55	0.51	0.50	0.48	0.51	0.47	0.42
C38	0.45	0.57	0.58	0.69	0.69	0.70	0.72	0.71	0.75	0.84	0.77	**1.00**	0.61	0.61	0.61	0.60	0.60	0.62	0.52	0.53	0.49	0.50	0.50	0.43
C44	0.47	0.59	0.57	0.68	0.69	0.73	0.76	0.71	0.74	0.74	0.73	0.76	**1.00**	0.73	0.61	0.59	0.69	0.60	0.50	0.53	0.49	0.51	0.49	0.44
C46	0.44	0.58	0.58	0.70	0.70	0.75	0.74	0.74	0.73	0.74	0.72	0.76	0.84	**1.00**	0.64	0.58	0.67	0.60	0.50	0.52	0.51	0.50	0.49	0.44
MIN	0.46	0.59	0.57	0.72	0.72	0.70	0.70	0.73	0.79	0.76	0.73	0.76	0.76	0.78	**1.00**	0.63	0.64	0.63	0.53	0.52	0.49	0.50	0.50	0.45
DLN	0.46	0.58	0.58	0.67	0.71	0.69	0.70	0.72	0.75	0.75	0.72	0.75	0.75	0.73	0.78	**1.00**	0.61	0.58	0.54	0.56	0.51	0.52	0.53	0.45
SIN	0.44	0.61	0.58	0.67	0.72	0.72	0.76	0.72	0.74	0.73	0.73	0.75	0.81	0.80	0.78	0.76	**1.00**	0.66	0.57	0.57	0.54	0.55	0.53	0.47
WOL	0.45	0.58	0.57	0.67	0.71	0.72	0.68	0.70	0.73	0.76	0.71	0.77	0.75	0.75	0.78	0.74	0.80	**1.00**	0.58	0.58	0.55	0.55	0.54	0.46
GOL	0.43	0.60	0.56	0.60	0.65	0.64	0.64	0.64	0.70	0.70	0.67	0.68	0.67	0.67	0.69	0.70	0.73	0.73	**1.00**	0.66	0.70	0.61	0.60	0.53
HER	0.44	0.59	0.56	0.63	0.66	0.66	0.67	0.65	0.69	0.71	0.67	0.69	0.69	0.68	0.69	0.72	0.72	0.73	0.80	**1.00**	0.67	0.62	0.62	0.52
ISK	0.40	0.58	0.56	0.61	0.64	0.63	0.65	0.62	0.67	0.68	0.65	0.66	0.66	0.67	0.66	0.68	0.70	0.71	0.83	0.80	**1.00**	0.62	0.62	0.54
ROK	0.41	0.59	0.57	0.62	0.65	0.65	0.68	0.65	0.66	0.66	0.68	0.66	0.68	0.66	0.67	0.68	0.71	0.71	0.76	0.77	0.77	**1.00**	0.67	0.58
SPT	0.42	0.57	0.54	0.64	0.65	0.63	0.66	0.65	0.67	0.67	0.64	0.67	0.65	0.66	0.66	0.69	0.69	0.70	0.75	0.76	0.77	0.80	**1.00**	0.61
ZOL	0.39	0.56	0.51	0.59	0.59	0.62	0.62	0.61	0.61	0.60	0.59	0.60	0.61	0.62	0.62	0.62	0.64	0.63	0.70	0.68	0.71	0.73	0.75	**1.00**

R-ISSR

Jaccard

**Nei**	L7661	L7662	L7987	BRA	CZA	ZIE	WOJ	ATU	DUE	JOL	Cl2	C38	C44	C46	MIN	DLN	SIN	WOL	GOL	HER	ISK	ROK	SPT	ZOL
*L*7661	**1.00**	0.29	0.26	0.35	0.35	0.36	0.35	0.34	0.34	0.33	0.37	0.32	0.33	0.34	0.34	0.35	0.36	0.33	0.31	0.34	0.32	0.31	0.34	0.33
*L*7662	0.45	**1.00**	0.56	0.54	0.52	0.51	0.53	0.52	0.51	0.51	0.51	0.54	0.52	0.50	0.46	0.50	0.52	0.52	0.52	0.55	0.55	0.51	0.51	0.45
*L*7987	0.41	0.72	**1.00**	0.55	0.54	0.53	0.52	0.50	0.50	0.52	0.46	0.51	0.55	0.49	0.49	0.50	0.52	0.48	0.47	0.44	0.46	0.41	0.45	0.44
BRA	0.51	0.70	0.71	**1.00**	0.89	0.79	0.78	0.72	0.77	0.67	0.70	0.69	0.71	0.71	0.69	0.65	0.65	0.70	0.65	0.63	0.65	0.57	0.62	0.63
CZA	0.51	0.69	0.70	0.94	**1.00**	0.82	0.83	0.72	0.74	0.69	0.66	0.69	0.73	0.73	0.71	0.69	0.65	0.71	0.67	0.64	0.66	0.58	0.63	0.63
ZIE	0.53	0.67	0.69	0.88	0.90	**1.00**	0.83	0.76	0.72	0.67	0.66	0.67	0.79	0.79	0.70	0.71	0.68	0.66	0.67	0.64	0.65	0.59	0.63	0.61
WOJ	0.51	0.69	0.68	0.88	0.90	0.91	**1.00**	0.72	0.76	0.70	0.68	0.72	0.79	0.74	0.69	0.72	0.69	0.68	0.69	0.66	0.69	0.64	0.64	0.64
ATU	0.51	0.68	0.67	0.83	0.84	0.86	0.84	**1.00**	0.76	0.72	0.67	0.67	0.73	0.71	0.75	0.71	0.66	0.73	0.67	0.62	0.62	0.60	0.63	0.61
DUE	0.51	0.68	0.66	0.87	0.85	0.84	0.86	0.86	**1.00**	0.77	0.80	0.73	0.74	0.71	0.74	0.69	0.68	0.78	0.69	0.62	0.65	0.59	0.63	0.62
JOL	0.50	0.67	0.68	0.81	0.82	0.80	0.82	0.84	0.87	**1.00**	0.72	0.76	0.71	0.70	0.71	0.68	0.65	0.68	0.67	0.61	0.64	0.59	0.64	0.59
C22	0.54	0.68	0.63	0.82	0.80	0.80	0.81	0.81	0.89	0.83	**1.00**	0.75	0.68	0.70	0.68	0.65	0.64	0.71	0.63	0.61	0.63	0.60	0.62	0.59
C38	0.49	0.70	0.68	0.82	0.82	0.81	0.84	0.80	0.85	0.87	0.85	**1.00**	0.74	0.72	0.68	0.68	0.71	0.67	0.71	0.67	0.67	0.61	0.62	0.61
C44	0.49	0.68	0.71	0.83	0.84	0.88	0.88	0.85	0.85	0.83	0.81	0.85	**1.00**	0.87	0.76	0.81	0.75	0.71	0.72	0.63	0.65	0.61	0.62	0.62
C46	0.51	0.67	0.66	0.83	0.84	0.88	0.85	0.83	0.83	0.83	0.82	0.84	0.93	**1.00**	0.78	0.78	0.73	0.70	0.71	0.64	0.67	0.62	0.64	0.64
MIN	0.50	0.63	0.66	0.82	0.83	0.83	0.82	0.85	0.85	0.83	0.81	0.81	0.86	0.88	**1.00**	0.74	0.72	0.75	0.69	0.66	0.66	0.61	0.67	0.65
DLN	0.52	0.67	0.66	0.79	0.82	0.83	0.84	0.83	0.82	0.81	0.78	0.81	0.89	0.88	0.85	**1.00**	0.81	0.74	0.76	0.69	0.68	0.65	0.69	0.67
SIN	0.53	0.68	0.68	0.79	0.79	0.81	0.82	0.80	0.81	0.78	0.78	0.83	0.86	0.84	0.84	0.89	**1.00**	0.72	0.77	0.73	0.72	0.66	0.67	0.66
WOL	0.50	0.68	0.65	0.83	0.83	0.80	0.81	0.84	0.88	0.81	0.83	0.81	0.83	0.82	0.85	0.85	0.84	**1.00**	0.73	0.69	0.71	0.61	0.66	0.65
GOL	0.47	0.68	0.64	0.79	0.80	0.81	0.81	0.80	0.82	0.80	0.77	0.83	0.84	0.83	0.82	0.86	0.87	0.84	**1.00**	0.75	0.82	0.70	0.74	0.72
HER	0.50	0.71	0.61	0.77	0.78	0.78	0.79	0.77	0.77	0.76	0.76	0.80	0.78	0.78	0.80	0.82	0.84	0.82	0.86	**1.00**	0.84	0.78	0.78	0.74
ISK	0.48	0.71	0.63	0.79	0.80	0.78	0.82	0.77	0.79	0.78	0.77	0.80	0.79	0.80	0.80	0.81	0.84	0.83	0.90	0.91	**1.00**	0.80	0.84	0.81
ROK	0.48	0.67	0.58	0.73	0.74	0.74	0.78	0.75	0.74	0.74	0.75	0.76	0.76	0.76	0.76	0.79	0.79	0.76	0.83	0.88	0.89	**1.00**	0.79	0.74
SPT	0.51	0.68	0.62	0.76	0.77	0.78	0.78	0.77	0.77	0.78	0.76	0.77	0.77	0.78	0.80	0.82	0.80	0.80	0.85	0.88	0.91	0.88	**1.00**	0.86
ZOL	0.50	0.62	0.61	0.77	0.77	0.76	0.78	0.76	0.76	0.74	0.74	0.76	0.76	0.78	0.79	0.80	0.80	0.79	0.84	0.85	0.89	0.85	0.92	**1.00**
